# A ligand discovery toolbox for the WWE domain family of human E3 ligases

**DOI:** 10.1038/s42003-024-06584-w

**Published:** 2024-07-24

**Authors:** Lena Münzker, Serah W. Kimani, Milan M. Fowkes, Aiping Dong, Hong Zheng, Yanjun Li, Morgan Dasovich, Krzysztof M. Zak, Anthony K. L. Leung, Jonathan M. Elkins, Dirk Kessler, Cheryl H. Arrowsmith, Levon Halabelian, Jark Böttcher

**Affiliations:** 1 Boehringer Ingelheim RCV GmbH & Co KG, Vienna, Austria; 2 Structural Genomics Consortium, University of Toronto, Toronto, ON Canada; 3 Princess Margaret Cancer Centre, Toronto, ON Canada; 4 Centre for Medicines Discovery, Nuffield Department of Medicine, University of Oxford, Oxford, UK; 5 Johns Hopkins University, Baltimore, MD USA; 6 Department of Medical Biophysics, University of Toronto, Toronto, ON Canada; 7 Department of Pharmacology and Toxicology, University of Toronto, Toronto, ON Canada

**Keywords:** X-ray crystallography, NMR spectroscopy, Small molecules

## Abstract

The WWE domain is a relatively under-researched domain found in twelve human proteins and characterized by a conserved tryptophan-tryptophan-glutamate (WWE) sequence motif. Six of these WWE domain-containing proteins also contain domains with E3 ubiquitin ligase activity. The general recognition of poly-ADP-ribosylated substrates by WWE domains suggests a potential avenue for development of Proteolysis-Targeting Chimeras (PROTACs). Here, we present novel crystal structures of the HUWE1, TRIP12, and DTX1 WWE domains in complex with PAR building blocks and their analogs, thus enabling a comprehensive analysis of the PAR binding site structural diversity. Furthermore, we introduce a versatile toolbox of biophysical and biochemical assays for the discovery and characterization of novel WWE domain binders, including fluorescence polarization-based PAR binding and displacement assays, ^15^N-NMR-based binding affinity assays and ^19^F-NMR-based competition assays. Through these assays, we have characterized the binding of monomeric *iso*-ADP-ribose (*iso*-ADPr) and its nucleotide analogs with the aforementioned WWE proteins. Finally, we have utilized the assay toolbox to screen a small molecule fragment library leading to the successful discovery of novel ligands targeting the HUWE1 WWE domain.

## Abstract (web summary)

Six WWE domain-containing proteins also contain domains with E3 ubiquitin ligase activity. We report structures, biophysical and biochemical assays for the discovery of novel WWE domain binders and their respective application.

## Introduction

**Fig. 1:**
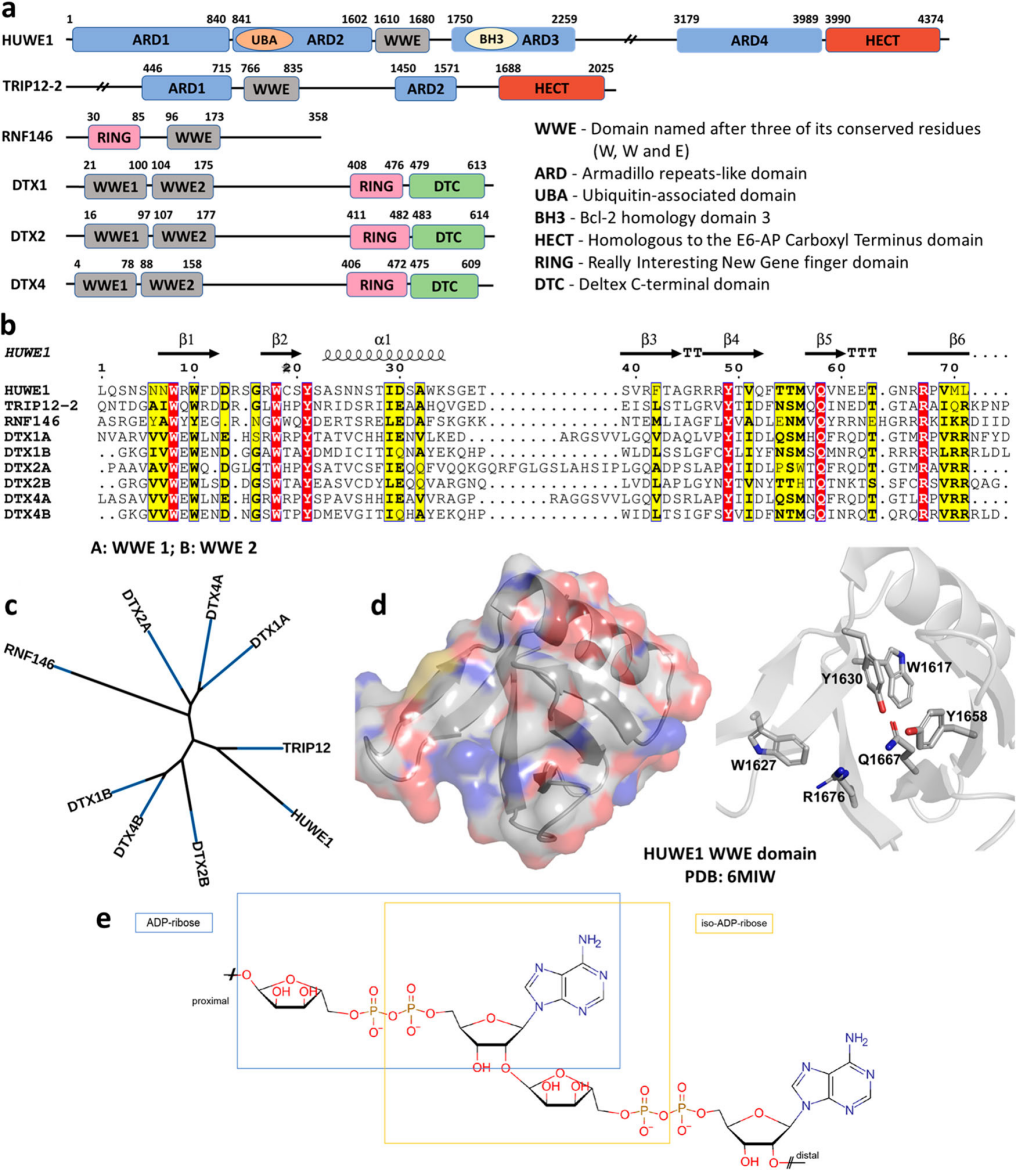
The WWE domains of human E3 ubiquitin ligases. **a** Domain architecture of the six known WWE domain-bearing human E3 ligases, derived from the available structures and AlphaFold prediction models. **b** Sequence alignment and **c** the phylogenetic tree of the WWE domains in the six human E3 ligases. The tree is displayed without considering branch lengths. The sequence of TRIP12 isoform 2 (Uniprot ID: Q14669-2) was used in the alignment. **d** The structure of the HUWE1 WWE domain (PDB ID: 6MIW) showing the overall fold (left) and the strictly conserved residues (sticks) corresponding to red-highlighted residues in the sequence alignment presented in panel **c**. Most of the strictly conserved residues are in the ADPr/PAR binding site. **e** Structural units of PAR. ADPr, the PAR building block and *iso*-ADPr, the internal PAR structural unit containing the ribose-ribose glycosidic bond, are highlighted.

The WWE domain was identified as a globular protein domain through sequence analysis. It derived its name from the presence of its highly conserved residues, namely two tryptophans and one glutamate^1^ (Fig. 1a, b). Based on the sequence homology within the protein family (Fig. 1b), it has been classified into three distinct subgroups according to the other protein domains found in each gene. The first group (PARP7, PARP11, PARP12, PARP13, and PARP14) is characterized by either a single WWE domain or a tandem WWE domain followed by a C-terminal poly-ADP-ribose polymerase (PARP) domain^2^. PARP domains impart mono- or poly-ADP-ribosylation catalytic activity to these proteins^3^, with PARP13 being the only member with an inactive PARP catalytic domain^4^. The second group consists solely of the phospholipase DDHD2^5^ and the third and largest group that is the subject of this study includes Deltex1 (DTX1), Deltex2 (DTX2), Deltex4 (DTX4), HUWE1, TRIP12, and RNF146/Iduna (Fig. 1a–c). This group is characterized by the presence of a domain with E3 ligase activity^6^. Within the third group, there are further sub-divisions based on the specific type of E3 ligase domain present in the protein. The first sub-division includes DTX1, DTX2, DTX4, and RNF146 and is characterized by a RING (Really Interesting New Gene) E3 ligase domain. The second sub-division comprises HUWE1 and TRIP12, which share a HECT (Homologous to E6-AP Carboxyl Terminus) E3 ligase domain (Fig. 1a). E3 ligases play a crucial role in the proteasomal degradation pathway. They work in a cascade with E1 (ubiquitin-activating) and E2 (ubiquitin-conjugating) enzymes to catalyze poly-ubiquitination of target proteins leading to proteasomal recognition and protein degradation^7,8^. General interest in E3 ligases and their respective binders has surged due to their potential use as Proteolysis-Targeting Chimeras (PROTACs)^9^ for the degradation of previously deemed undruggable proteins. While there are more than 600 E3 ligases encoded in the human genome, the development of PROTACs has primarily focused on a subset of E3 ligases. Some of the E3 ligases that have been recently employed include Von-Hippel Lindau (VHL)^10^, cereblon (CRBN)^11^, and IAPs^12^, amongst others. Due to its role as a substrate recruitment domain within E3 ligases, the WWE domain may represent an attractive domain to employ as an E3 handle for PROTAC development. Here, we provide a short overview of the function and relevance of the six WWE-domain containing E3 ligase family members (Fig. 1c) and present a comprehensive sequence and structural analysis. We generated novel crystal structures of HUWE1, TRIP12, and DTX1 in the presence of nucleotides and developed a fluorescence-based PAR binding assay, NMR-based *K*_d_ determination assay, and ^19^F-based competition assay in conjunction with X-ray structural data analysis to offer a versatile toolbox for studying WWE-domain containing E3 ligases. This toolbox facilitates the identification of novel WWE ligands with the potential to expand the application of E3 ligases in the PROTAC approach with HUWE1, TRIP12, RNF146 and DTX1/2. Finally, a Fragment-Based Screen was employed to experimentally evaluate the ligandability of the WWE domain in HUWE1.

### HUWE1

HUWE1 is a 482 kDa essential enzyme with an expanding list of diverse substrates attributed to its various substrate recognition domains, which include a Bcl2-homology three (BH3) domain, a WWE domain, and Ubiquitin-associated (UBA) modules^13^ (Fig. 1a). HUWE1 features a C-terminal HECT domain with a catalytic cysteine that catalyzes mono-ubiquitination, K48-, K63- and K6-linked poly-ubiquitination for proteasomal degradation, protein regulation and cellular signal transduction^14^. Major cellular processes that are regulated by HUWE1’s activity include cell cycle control, autophagy, apoptosis, DNA damage repair, and inflammation through interactions with various proteins such as c-Myc^15^, Mcl1^16,17^, and p53^16^. In addition, HUWE1 functions as a quality control enzyme through degradation of unassembled components of multi-protein complexes, such as the ribosome^18^ and nucleosomes^17,19^.

Recently, two cryo-EM structures revealed that HUWE1 forms a large alpha solenoid structure composed of four armadillo repeat-like domains (ARLD) with an inner circumference of ~250 Å^13,20^. The WWE domain is located above the ring plane, opposite the catalytic HECT domain. The enzyme undergoes a significant conformational change from an inactive “open” conformation (also described as the T-state/E2-binding state) to a closed conformation essential for E3 ligase activity^20^.

### TRIP12

TRIP12 (Thyroid Hormone Receptor Interacting protein 12) is primarily a nuclear protein belonging to the HECT ubiquitin ligase family and promotes ubiquitination and degradation of the tumor suppressor protein ARF^21^. The full-length TRIP12 protein is composed of several structural domains including a catalytic HECT domain, protein–protein interaction domains like the WWE domain and Armadillo repeats (ARM), and an intrinsically disordered region (IDR) that interacts with chromatin^22^ and possibly microtubules^23^ (Fig. 1a). TRIP12 is involved in the regulation of key biological processes such as chromatin remodeling, DNA damage response, cell cycle progression and cell differentiation through ubiquitination-mediated degradation of key substrate proteins^21,23–27^. Alterations in TRIP12 expression resulting from mutations, amplifications, fusions, and deletions have been linked to various cancers, as reviewed by Brunet et al.^27^. According to The Cancer Genome Atlas (TCGA) Pan-Cancer analysis data on cBioPortal, approximately 4% of cancer patients exhibit TRIP12 alterations^28,29^. At the gene level, *TRIP12* gene modifications are associated with autism spectrum disorder (ASD) and intellectual disorders, including Clark-Baraitser syndrome^30,31^. TRIP12 has been shown to regulate PARP1 stability and turnover *via* its PAR-targeted ubiquitin ligase (PTUbL) activity. Specifically, the WWE domain of TRIP12 binds to PAR modifications on the PARylated PARP1 enzyme, triggering allosteric activation of its catalytic HECT domain. This leads to PARP1 ubiquitination and degradation^26^. In this context, TRIP12 has been found to reduce the sensitivity of cancer cells to PARP inhibitors (PARPi). The loss of TRIP12 restores sensitivity in a PARP1-dependent manner through enhanced PARP1 trapping^26^.

### RNF146

RNF146, also known as Iduna, is composed of 358 amino acids and is characterized by the presence of an *N-*terminal WWE domain and a RING domain^32^ (Fig. 1a). Its E3 ubiquitin ligase activity is poly-ADPr (PAR)-dependent, with allosteric activation of the E3 ligase RING domain upon PAR binding to the WWE domain^33^. The WWE domain specifically recognizes *iso*-ADPr, a structural subunit of PAR and is reported to bind with an affinity of 370 nM to it^6^. Mutations in the PAR binding interface eliminate RNF146’s E3 ligase activity^18,34^. This unique activation mechanism explains RNF146’s involvement in DNA repair mechanisms and various cellular functions *via* interactions with PARylated proteins, including Axin, a component of the β-catenin construction complex. Small Ubiquitin-Related Modifier(SUMO)ylation of RNF146 enhances Axin degradation and the dysregulation has adverse implications for cancer progression^35,36^. RNF146 also regulates Tankyrase-dependent ADP-ribosylated adapter protein SH3-domain binding protein 2 (3BP2)^37,38^.

### DTX1, DTX2, and DTX4

The Deltex proteins belong to the RING-H2 family of ubiquitin E3 ligases^39,40^. Humans possess five Deltex genes (DTX1, DTX2, DTX3, DTX3L, and DTX4), which control the differentiation of cells through ubiquitination, methylation, JNK signaling, and Wnt signaling^41^. Mutations in DTX1 have been connected to poorer survival rates in patients with diffuse large B-cell lymphoma^42^. In contrast to other family members, DTX1, DTX2, and DTX4 contain a pair of *N*-terminal WWE domains, and these are followed by a catalytic RING-H2 domain and the Deltex C-terminal (DTC) domain^40^ (Fig. 1a). Recently, progress has been made in understanding the function of the RING-H2 and DTC domains of DTX1 and DTX2^43–45^. Chatrin et al. found that the DTC domain of DTX1 binds to nicotinamide adenine dinucleotide (NAD^+^), a substrate that enables the ADP-ribosylation of ubiquitin recruited as E2~Ub by the RING-H2 domain, thereby blocking its activation^43^. Ahmed and co-workers showed that ubiquitination of DTX2 substrates in cell-based ubiquitination assays could proceed in the presence of the RING-H2 and DTC domain fragment alone^44^. Furthermore, the specific mechanism of ubiquitination *via* the RING-H2 and DTC domains of the Deltex family was recently determined to occur without an E3~Ub intermediate^45^. However, the role of the Deltex WWE domains in ubiquitination have not been described, despite DTX2 showing binding to PAR in vitro^44^.

## Results

### Protein production

To study the molecular interactions of the WWE domain with different ligands, we designed, cloned, expressed, and purified WWE domains of HUWE1 (Uniprot ID: Q7Z6Z7, residues 1611–1700), TRIP12-2 (Uniprot ID: Q14669, residues 759–847), RNF146 (Uniprot ID: Q9NTX7, residues 100-184), DTX1 (Uniprot ID: Q86Y01, residues 21–184) and DTX2 (Uniprot ID: Q86UW9, residues 3–189) (Table S1).

The design of the RNF146 and HUWE1 WWE domain constructs was based on the published WWE domain structure of RNF146 (PDB ID: 3V3L)^6^. For TRIP12, we initially expressed and purified several WWE domain constructs with varying domain boundaries from the isoform 1 sequence, but some of the proteins exhibited instability and none showed binding to PAR polymers in our Fluorescence Polarization (FP) binding assay. Further structural analysis of the AlphaFold-predicted TRIP12 WWE domain structure (isoform 1; protein ID: NP_004229.1) revealed that a critical region within the WWE domain was missing in TRIP12 isoform 1 (Fig. S1), but these residues are present in isoform 2 (protein ID: NP_001271144.1), which contains a 28-residue insertion at residue 784. Consequently, two TRIP12 WWE domain constructs of the isoform 2 sequence were generated for structural and interaction studies.

Unlike HUWE1, RNF146 and TRIP12 which each possess only one WWE domain, the DTX subfamily is characterized by a tandem-WWE arrangement. Initially, we conducted a sequence alignment of the WWE domains of *Drosophila* Deltex and human DTX1, DTX2, and DTX4 to look for conserved residues across species (Fig. S2). This analysis revealed that numerous residues were conserved between WWE1 and WWE2 domains within a species (e.g. DTX1B and DTX4B), suggesting that they may possess similar ligand-binding capacities. In addition, many residues were also conserved between human DTXs and *Drosophila* Dx (e.g. DTX1A and DTXA), implying that the WWE domains of both species might share a similar structure. Based on the sequence alignment, the AlphaFold-predicted structures and the structure of *Drosophila* Deltex^41^, we designed expression constructs for the human DTX1, DTX2, and DTX4 WWE domains. All proteins were expressed ^15^N labeled or unlabeled in *E. coli*, and protocols can be found in the supplementary information. The expression of stable DTX4 protein was unsuccessful in bacterial cells and thus, this protein was excluded from this study.

### 11-mer PAR fluorescence polarization (FP) binding assay

**Fig. 2:**
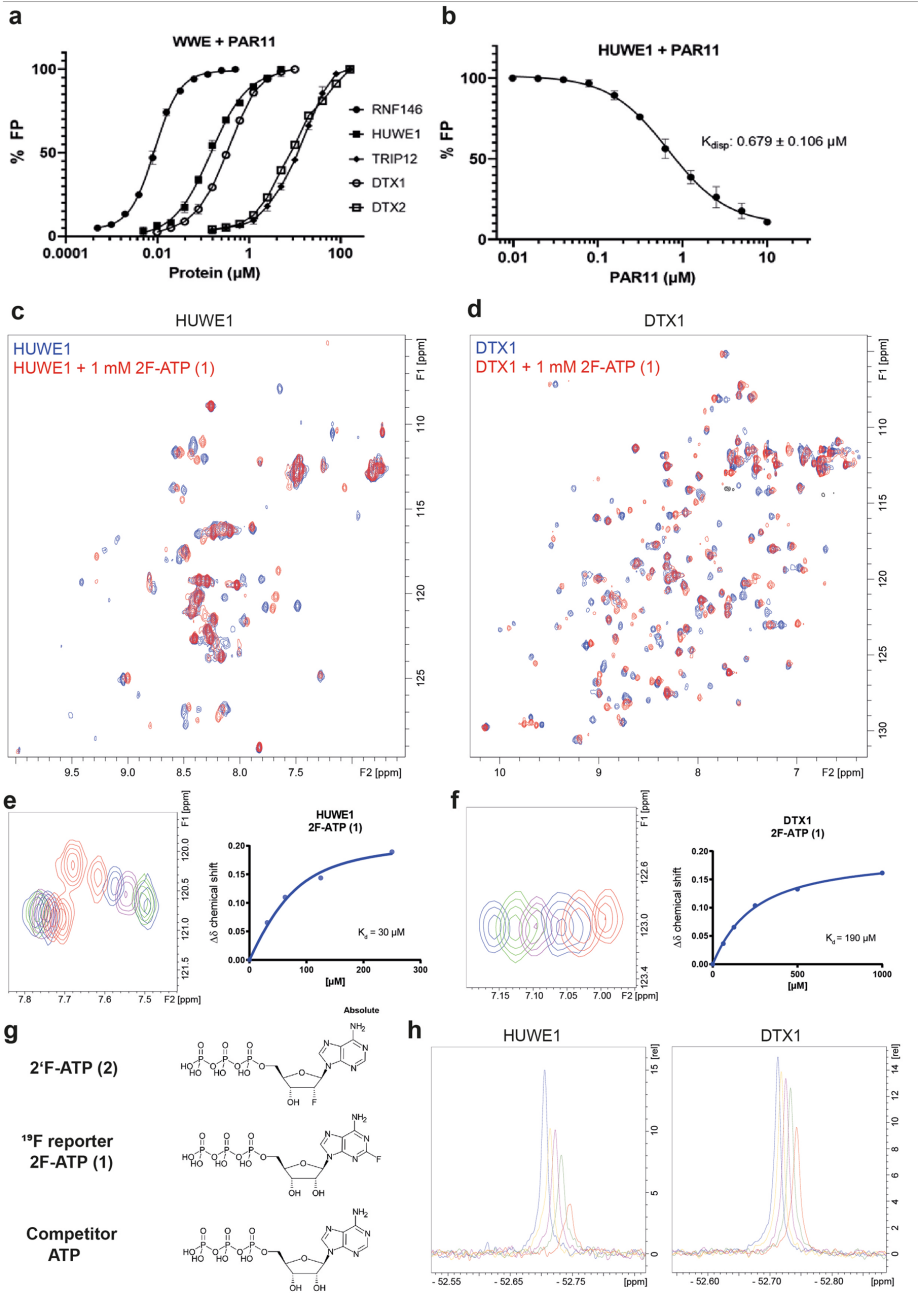
Binding interactions of PAR and ATP-analogs to the E3 ligase WWE domains. **a** Fluorescence-polarization-based binding of fluorescein-labeled 11-mer of PAR (FAM-PAR) to the WWE domains of HUWE1, RNF146, TRIP12, DTX1, and DTX2. Fluorescence polarization percentage (% FP) of the reference is plotted as a function of WWE protein concentration in µM using a logarithmic scale. **b** Competitive displacement of FAM-labeled 11-mer PAR from the HUWE1 WWE domain by an unlabeled 11-mer PAR. Fluorescence polarization percentage (% FP) of the reference is plotted as a function of unlabeled PAR 11-mer in µM using a logarithmic scale. For the FP assays, all experiments were conducted in triplicate with three experimental repeats for direct PAR binding to HUWE1, TRIP12, DTX1, and RNF146, and two experimental repeats for direct PAR binding to DTX2, as well as FAM-PAR displacement in HUWE1. Samples from each experimental repeat were processed independently to ensure reproducibility and minimize bias. The plotted values represent the averages from the experimental repeats, and the statistical analyses conducted using GraphPad Prism Version 9.1.0 represent the standard deviations resulting from the analyzed repeats. **c**, **d** ^15^N-HSQC spectra of **c** HUWE1 and **d** DTX1 overlaid with protein in the presence of 1 mM 2F-ATP (**1**). **e**, **f** ^15^N-HSQC NMR *K*_d_ titration assay and zoom-in on a peak upon a two-fold titration of 2F-ATP (**1**) to **e** HUWE1 and **f** DTX1 using a concentration range of 62.5 µM to 2 mM. NMR *K*_d_ values originate from distinct samples (*n* = 1) measured for each concentration, mean *K*_d_s are obtained from curves of selected cross peaks ± standard deviations, ligand concentrations are plotted on the x-axis and the Δδ chemical shifts on the y-axis. **g** Chemical structure of ^19^F reporter 2F-ATP (**1**) 2'F-ATP (**2**) and competitor (ATP). **h** ^19^F-Displacement assay with HUWE1 and DTX1. In red lines, the ^19^F-NMR of 2F-ATP (**1**) reporter in the presence of protein is shown. In green, brown, and yellow, the titration of ATP at 250 mM, 500 mM, and 1000 mM concentration, respectively, to the protein in presence of 2F-ATP (**1**) is plotted. In blue lines, the ^19^F-NMR of the reporter 2F-ATP (**1**) reporter without protein is shown. ^19^F signals are displayed at an offset of 0.01 ppm to enhance clarity.

FP is a versatile technique that is extensively used in molecular interactions assays including small molecule screening^46^. To ensure broad application within the WWE domain family, we prepared a Fluorescein amidite (FAM) labeled linear 11-mer PAR substrate, inspired by the physiological PAR polymers that are recognized by the WWE domains. The assay was initially applied to the single WWE domains of HUWE1, RNF146, and TRIP12 (Fig. 2a, b; Table 1). Both RNF146 and HUWE1 demonstrated nanomolar binding to 11-mer PAR, with *K*_d_ values of 8.7 nM and 150 nM, respectively. Conversely, TRIP12 exhibited weaker binding to 11-mer PAR, with a *K*_d_ value of 13.3 µM. The E3 ligases DTX1, DTX2, and DTX4 possess a pair of tandem WWE domains, which exhibit conservation of key residues in the ligand-binding site (Fig. 1). Given that some of these ligases bind PARylated substrates in cells^44^, we also expected PAR binding in vitro. Indeed, DTX1 showed nanomolar binding to 11-mer PAR with a *K*_d_ value of 340 nM, while DTX2 had a much lower affinity for 11-mer PAR with a *K*_d_ of 9.26 µM (Fig. 2a, b; Table 1)^44^. To ascertain the specificity of the FP assay and assess its potential utility in molecular screening applications, we designed an FP displacement assay using an unlabeled PAR 11-mer polymer. In this assay, the unlabeled PAR displaced 11-mer FAM-PAR binding in HUWE1 in a dose-dependent manner, with a *K*_disp_ value of 679 nM (Fig. 2b).

**Table 1:** Binding affinities of molecules to the human WWE domains

Binding affinities						
	PAR-derived molecules			ATP and analogs		
E3 ligase	*iso*-ADP-ribose^a^ *K*_d_ [µM]	(FAM) 11-mer PAR^b^ *K*_d_ [µM]	ADPr^a^ *K*_d_ [µM]	ATP^a^ *K*_d_ [µM]	2F-ATP 1^a^ *K*_d_ [µM]	2’F-ATP 2^a^ *K*_d_ [µM]
RNF146	<10	0.0087 ± 0.001	22 ± 1	352 ± 91	49 ± 18	114 ± 31
TRIP12	>500	13.3 ± 1.20	118 ± 45	316 ± 22	127 ± 9	125 ± 20
HUWE1	132 ± 1	0.15 ± 0.015	31 ± 6	59 ± 5	27 ± 7	15 ± 2
DTX1	190 ± 32	0.34 ± 0.005	709 ± 237	659 ± 294	329 ± 115	550 ± 111
DTX2	−	9.26 ± 2.36	314 ± 40	802 ± 84	>2000	455 ± 17

^a^Determined with ^15^N-HSQC-NMR titrations, NMR *K*_d_ values originate from distinct samples (*n* = 1) measured for each concentration, mean *K*_d_s were obtained from curves of selected cross peaks ± standard deviations.

^b^Determined from titrations of Fluorescein amidite (FAM) labeled linear 11-mer PAR in 3 distinct triplicates (*n* = 3), with two to three experimental repeats for each protein. Mean *K*_d_ values were obtained from curves constructed with average values of the experimental repeats ± standard deviations.

Depending on the substrate specificity of each WWE domain tested here, there is a possibility for more than one WWE domain molecule to bind to a single linear 11-mer PAR chain in our assay. Recent work on the PARP13 tandem WWE domains revealed that only one of the domains is functional, and it preferentially binds to the terminal end of the PAR chains^47^. Whether E3 WWE domains exhibit such a distinctive mode of PAR recognition is still unknown.

### Isotopic labeling and ^1^H^15^N-HSQC NMR assays

Protein-observed NMR using isotopically labeled proteins has emerged as one of the most sensitive and widely employed methods for fragment-based screening^48^. Our objective was to generate ^15^N-labeled proteins of WWE domain family members, thereby enabling ^15^N-HSQC NMR studies. This approach provides a protein-based fragment screening assay to identify and orthogonally validate binders for the WWE domain family proteins. We conducted NMR-based *K*_d_ determination assays for HUWE1, DTX1, RNF146, TRIP12, and DTX2 (Fig. 2c–f, Fig. S3). The small size of the WWE domain constructs (10–20 kDa) yielded NMR spectra with well-resolved individual peaks when analyzed on a 600 MHz spectrometer. This facilitated the determination of *K*_d_ values for ligands in the fast-exchange regime by monitoring chemical shift perturbations in the ligand titration. We tested the binding of endogenous nucleotide-based ligands, including *iso*-ADPr, ADPr, and ATP to the WWE domains of RNF146, TRIP12, HUWE1, DTX1, and DTX2 (Fig. 2c–f, Fig. S3–S7). ATP was selected as fluorinated analogs were available for later usage as displacement probes, namely 2F-ATP (**1**) and 2’F-ATP (**2**), which were also included in the titrations. The adenosine-based ligands were titrated in the range of 62.5 µM to 2 mM for each WWE domain protein. However, due to resource intensive preparation, *iso*-ADPr was only titrated up to 500 µM. We then plotted the Δδ chemical shifts and ligand concentrations to determine a saturation curve. For each *K*_d_ determination, the chemical shifts of at least two separate peaks were analyzed and reported as the mean with standard deviation (Table 1, Figs. S4–7).

The *K*_d_ determination was initiated with RNF146, given its status as the most extensively studied E3 ligase. Previous reports have indicated that *iso*-ADPr binds to RNF146 with a dissociation constant of 0.37 µM^6^. Consistent with these findings, our results showed a *K*_d_ of <10 µM, representing the limit of our assay due to the protein concentration utilized (Table 1). However, the affinity of the PAR-derived ADPr was relatively weak, with a *K*_d_ of 22 µM. In contrast, ATP and the ATP analogs we tested, namely 2F-ATP (**1**) and 2’F-ATP (**2**) exhibited affinities ranging from 49 µM to 352 µM (Table 1). Differences to previously reported affinities determined by ITC^49^ can likely be attributed to deviating experimental setups and detection methods. Unlike the dissociation constant of *iso-*ADPr for RNF146, the dissociation constant of *iso-*ADPr for TRIP12 was outside the titration range tested (*K*_d_ > 500 µM). ADPr bound with an affinity of 118 µM, while ATP’s dissociation constant was 316 µM. Similarly, the two F-ATP ligands for TRIP12, 2F-ATP (**1**) and 2’F-ATP (**2**) were detected with a *K*_d_ of 127 µM and 125 µM, respectively (Table 1). Overall, the binding data indicates that TRIP12 exhibits weaker binding to PAR polymers and isolated nucleotide analogs. Generally, the dissociation constants of our ligands for HUWE1 were higher, apart from *iso-*ADPr, which was determined to be the weakest (*K*_d_ = 132 µM, compared to ADPr, ATP, 2F-ATP (**1**) and 2’F-ATP (**2**) with *K*_d_’s of 15 µM to 59 µM) (Table 1).

In general, the binding of ADPr and ATP to DTX1 was found to be very weak, with affinities of 709 μM and 659 μM, respectively. The binding affinities of DTX2 for both ligands were similar to those observed for DTX1, with only a marginal improvement in binding affinity for ADPr (*K*_d_ = 314). The binding of the *iso-*ADPr subunit of PAR to DTX1 exhibited the lowest *K*_d_ value (190 μM), however, this affinity was still considerably weaker in comparison to the binding of the same subunit to the RNF146 WWE domain (*K*_d_ < 10 µM) (Table 1). Owing to the restricted availability of *iso*-ADPr, we solely titrated *iso*-ADPr with DTX1. In summary, the binding data reveals that the WWE domains of both DTX1 and DTX2 display the weakest binding affinities for the examined ligands in comparison to the other E3 ligases.

### ^19^F NMR displacement assay

Based on the protein-observed *K*_d_ determinations, we developed a ^19^F-NMR assay using the above validated binder (2F-ATP (**1**)) as a reporter. Displacement with an excess of nonfluorinated compound binding in the same binding pocket as the ligand of interest provides a rapid assay for competition in the same binding site^50^. For 2F-ATP (**1**), a single peak signal was recorded in the ^19^F-NMR spectrum, and its chemical shift and intensity changes when bound to the protein. For controls, we measured 2F-ATP (**1**) both with and without protein and added increasing amounts of ATP up to 1 mM. For all tested WWE domain proteins, competition between ATP and 2F-ATP (**1**) in the same binding site resulted in increasing signal intensity, reflecting the increasing proportion of free (unbound) 2F-ATP (**1**) ^19^F-ligand (Fig. 2g, h and Fig. S8). We optimized the protein concentration for each E3 ligase WWE domain to achieve a large signal intensity difference window between 2F-ATP (**1**) only and 2F-ATP (**1**) with protein. However, F-ATP binding affinities varied, ranging from 27 µM for HUWE1 to >2 mM for DTX2, while ATP binding affinities ranged from 59 µM for HUWE1 to 802 µM for DTX2 (Table 1). Generally, ATP binding affinities were twice as weak as those of 2F-ATP (**1**), except for RNF146, which exhibited a six-fold weaker affinity for ATP (Table 1). In the case of DTX2, the assay was not successful as the affinity of ATP (802 µM) was too weak for displacement of 2F-ATP (**1**) (Figure S8).

### Structural characterization of all human E3 WWE domains interaction with PAR building units and analogs

**Fig. 3:**
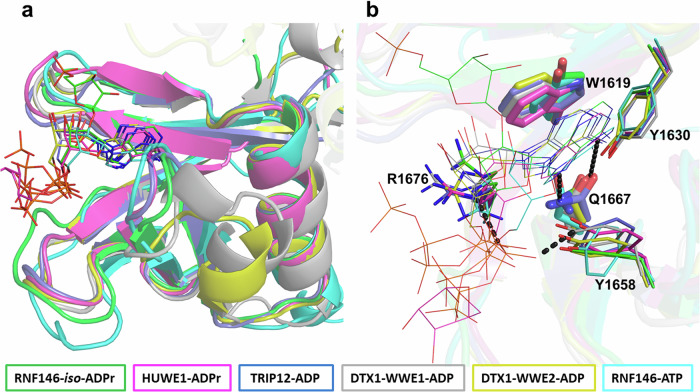
The WWE domain fold conservation in our natural ligands-complexed structures. **a** A superposition of the reported RNF146-*iso*-ADPr structure (3V3L; green), the mouse RNF146-ATP structure (2RSF; ensemble 1; cyan) and the structures of HUWE1-ADPr (magenta), TRIP12-ADP (slate blue), DTX1-WWE1-ADP (gray), and DTX1-WWE2-ADP (yellow). The ligands are shown as thin sticks. **b** A close-up view of the ligand-binding site in the superimposed structure in panel **a** showing the conserved interaction of the ligands (lines) with the strictly conserved residues (thick and thin sticks). Residues are labeled based on HUWE1 WWE domain and the potential hydrogen bonds in the HUWE1-ADPr are shown as black dashes.

RNF146 is the only human E3 ligase WWE domain for which the molecular recognition of a PAR structural unit has been reported in detail, aided by the co-crystal structure of the domain in complex with *iso*-ADPr (PDB ID: 3V3L)^6^. To support future drug-discovery efforts and ultimately increase the structural coverage of the WWE domain family, we determined the crystal structures of the WWE domains of HUWE1, isoform 2 TRIP12 and Deltex 1 in complex with a selection of PAR building blocks and their analogs, as well as HUWE1 in complex with two compounds identified using the tools developed in this study (Tables 2 and 3). The omit maps of the ligands of interest in our structures are shown in Fig. S9. The overall WWE fold of six β-strands forming half a β-barrel covered by an α-helix was highly conserved in our solved structures and overlapped well with the reported RNF146 WWE structures^6,49^ (Fig. 3a and Table S2). In addition to structural conservation, the DTX1-ligand complexed structures had the tandem WWE domains joined to each other by a short linker as in the structure of *Drosophila* Dx^41^ (Fig. S10).

**Table 2:** Data collection and refinement statistics part 1

	HUWE1- ADPr	HUWE1- 2’F-ATP (2)	TRIP12- ATP	TRIP12- ADP	DTX1- ATP
PDB ID	8RD7	8R7O	9BKR	9BKS	8R5N
**Data collection**					
Space group	P4_3_ 2_1_ 2	P4_3_2_1_2	P2_1_2_1_2_1_	P2_1_2_1_2_1_	P 2_1_
Cell dimensions					
* a*, *b*, *c* (Å)	62.9, 62.9, 231.2	62.9, 62.9, 231.9	30.4, 33.5, 66.1	30.4, 33.7, 66.4	67.9, 33.9, 84.0
α, β, γ (°)	90, 90, 90	90, 90, 90	90, 90, 90	90, 90, 90	90, 90, 90
Resolution (Å)	60.7–1.3 (1.44–1.32)^a^	60.7–1.36 (1.45–1.36)^a^	55.0–1.40 (1.42–1.40)^a^	50.0–1.17 (1.19–1.17)^a^	84.04–1.81 (1.84–1.81)^a^
*R*_sym_ or *R*_merge_	0.057 (1.54)^a^	0.060 (2.336)^a^	0.134 (0.843)^a^	0.057 (0.251)^a^	0.130 (3.200)^a^
*I* / σ*I*	20.5 (1.5)^a^	23.2 (1.4)^a^	29.37 (1.97)^a^	41.8 (4.96)^a^	6.3 (0.8)^a^
Completeness (%)	95.3 (61.5)^a,b^	95.9 (61.5)^a,b^	97.9 (78.2)^a,b^	98.3 (83.0)^a^	97.6 (96.6)^a^
Redundancy	12.9 (12.3)^a^	22.0 (22.8)^a^	12.2 (4.5)^a^	6.6 (3.3)^a^	6.4 (6.2)^a^
**Refinement**					
Resolution (Å)	1.31	1.36	1.40	1.17	1.81
No. reflections	87,482	86,990	12,941	22,138	34,305
*R*_work_/*R*_free_	14.8/17.7	16.8/19.5	16.2/20.8	14.9/17.3	17.3/21.9
No. atoms					
Protein	2529	2504	614	648	2730
Ligand/ion	51	66	31	30	62
Water	435	376	48	75	153
*B*-factors					
Protein	26.4	32.3	21.9	11.2	43.7
Ligand/ion	35.7	56.3	21.3	12.3	50.2
Water	42.1	42.6	32.5	22.8	51.1
R.m.s. deviations					
Bond lengths (Å)	0.012	0.012	0.008	0.007	0.091
Bond angles (°)	1.29	1.21	1.36	1.49	1.45

One crystal was used to obtain the structures.

^a^Values in parentheses are for highest resolution shell.

^b^Completeness is ellipsoidal as output by staraniso.

**Table 3:** Data collection and refinement statistics part 2

	DTX1-WWE1- ADP	DTX1-WWE2- ADP	HUWE1- Compound (3)	HUWE1- Compound (4)
PDB ID	8R6A	8R6B	8RD0	8RD1
**Data collection**				
Space group	P2_1_	P2_1_2_1_2_1_	P4_3_2_1_2	P4_3_2_1_2
Cell dimensions				
* a*, *b*, *c* (Å)	68.1, 34.1, 84.6	67.4, 70.3, 33.9	62.8, 62.8, 231.8	62.8, 62.8, 231.5
α, β, γ (°)	90, 90, 90	90, 90, 90	90, 90, 90	90, 90, 90
Resolution (Å)	84.578–2.4 (2.45–2.40)^a^	35.147–2.5 (2.60–2.50)^a^	60.61–1.77 (1.86–1.77)^a^	60.58–1.89 (1.92–1.89)^a^
*R*_sym_ or *R*_merge_	0.122 (0.701)^a^	0.115 (0.242)^a^	0.172 (2.57)^a^	0.135 (1.171)^a^
*I* / σ*I*	7.3 (1.5)^a^	10.8 (4.0)^a^	13.4 (1.5)^a^	13.4 (1.7)^a^
Completeness (%)	95.3 (96.7)^a^	92.9 (77.4)^a^	95.1 (51.6)^a,b^	93.2 (28.8)^a^
Redundancy	2.3 (2.3)^a^	5.9 (3.9)^a^	23 (23.5)^a^	12.7 (8.1)^a^
**Refinement**				
Resolution (Å)	2.4	2.5	1.76	1.89
No. reflections	15,032	5483	42,066	35,344
*R*_work_/*R*_free_	27.1/30.9	22.1/25.0	0.197/21.5	17.9/22.0
No. atoms				
Protein	2707	1370	2496	2498
Ligand/ion	54	27	36	59
Water	111	5	380	378
*B*-factors				
Protein	41.3	16.0	32.3	31.1
Ligand/ion	53.8	33.0	34.9	35.6
Water	37.9	16.5	45.7	45.7
R.m.s. deviations				
Bond lengths (Å)	0.003	0.003	0.009	0.092
Bond angles (°)	0.74	0.72	0.88	1.16

One crystal was used to obtain the structures.

^a^Values in parentheses are for highest resolution shell.

^b^Completeness is ellipsoidal as output by staraniso.

The WWE domain is characterized by conserved residues (Fig. 1b), some of which interact directly with ligands, while others play structural stabilization roles. A highly conserved feature in all determined structures is the recognition and therefore the interactions involving the adenine ring of the ligands. The side chain of a highly conserved glutamine forms a dual hydrogen bond with the adenine ring of the substrate-derived ligands in all structures generated in this study, as well as in previously reported RNF146:*iso*-ADPr^6^ and RNF146-ATP^49^ structures (Fig. 3b). Furthermore, the adenine ring is stabilized through *π-π* interactions with the aromatic ring of a tryptophan, which is conserved across all E3 WWE domain proteins except for RNF146, where it is substituted by a tyrosine, whose side chain fulfils an analogous function (Fig. 3b). The interactions involving the proximal phosphate group are also conserved across all structures, where two key hydrogen bonds to the sidechains of conserved tyrosine and arginine residues are observed (Fig. 3b). The ribose and the distal phosphate moieties of the ligands interact with polar and positively charged regions within the three middle β-strands—and these regions appear to play a role in defining ligand specificity.

The RNF146 WWE domain is a potent binder of 11-mer PAR polymers (Fig. 2a; Table 1) and prefers *iso*-ADPr to ADPr in both applied assays (Table 1) and a previous study by Wang et al.^6^. The *iso*-ADPr specificity has been attributed to a hydrogen bonding interaction between the hydroxyl group of Tyr107 and the distal ribose oxygen of *iso*-ADPr as well as extended charged interactions with the distal phosphate (Fig. S11a), which is in line with affinity losses for *iso*-ADPr following respective binding site mutations^6^. Specifically, the distal phosphate is tightly encased in a positively charged *β*-loop-*β* region at one edge of the half β-barrel (residues 107–114), interacting with Arg110 and Trp114, as well as Lys175 belonging to a C-terminal loop outside the WWE domain (Fig. S11a).

### The structures of HUWE1 in complex with ADPr and TRIP12 with ADP

**Fig. 4:**
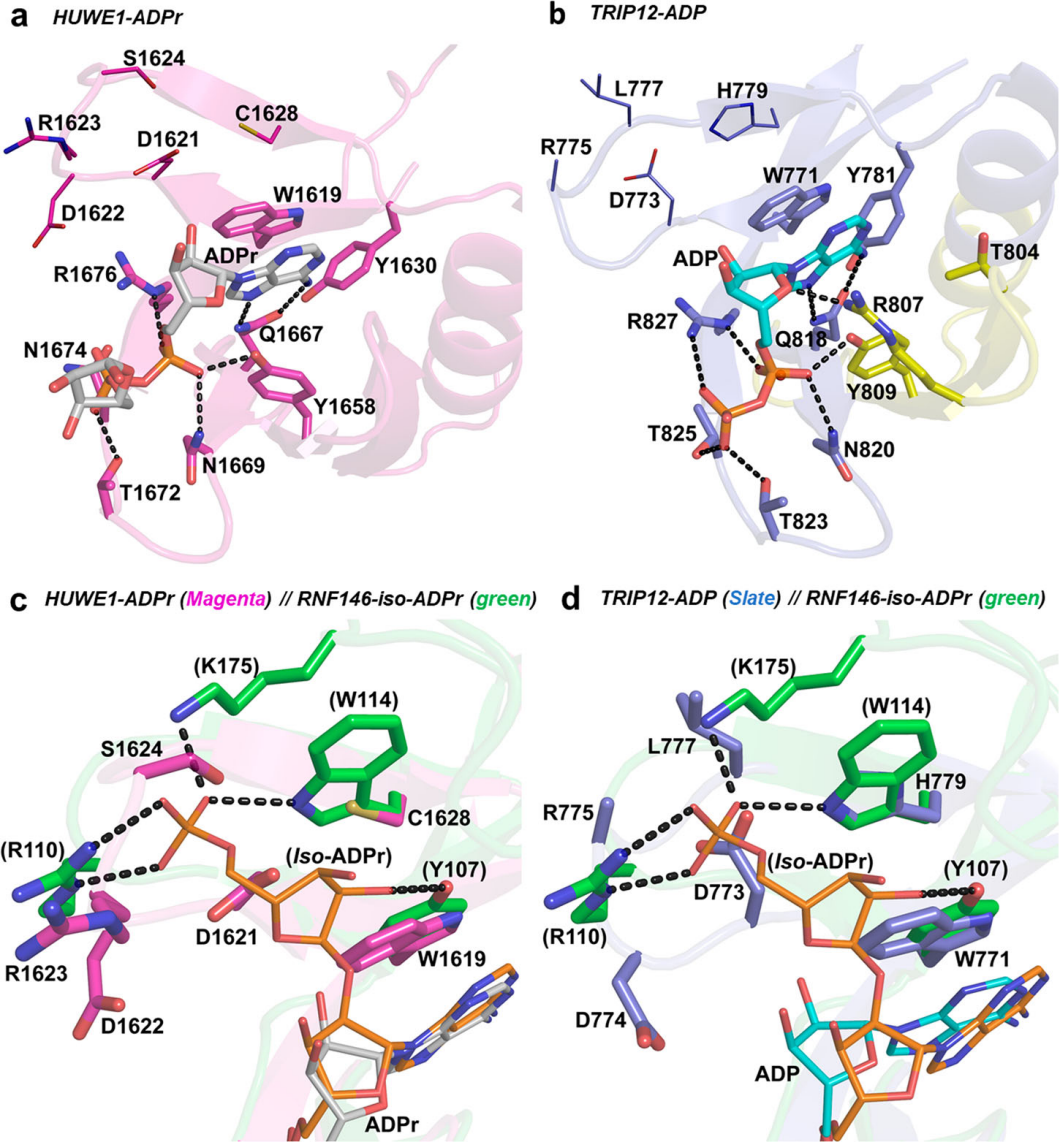
The WWE domain ligand-binding sites in TRIP12 and HUWE1. **a** The ligand-binding site of the HUWE1 WWE domain bound to ADPr (gray sticks). Residues interacting with ADPr are shown as sticks and potential hydrogen bonds are shown as black dashes. Residues depicted as lines correspond to the binding site of the distal phosphate group of *iso*-ADPr as in the RNF146-*iso*-ADPr structure (PDB: 3V3L). **b** The ligand-binding site of the isoform 2 TRIP12 WWE domain bound to ADP. The protein is shown in slate blue, with a yellow section representing 28 residues that are missing within the WWE domain of the canonical isoform 1 TRIP12 sequence (Uniprot ID: Q14669-1). ADP is shown as cyan sticks, ADP-interacting residues as shown as sticks and potential hydrogen bonds are depicted as black dashes. Similarly, residues depicted as lines correspond to the binding site of the terminal phosphate group of *iso*-ADPr as in the RNF146-iso-ADPr structure (PDB: 3V3L). **c** Superposition of the HUWE1-ADPr WWE domain (magenta) with *iso*-ADPr bound RNF146 WWE domain (green; PDB: 3V3L), showing the differences in the binding site of the distal ribose and phosphate groups of *iso*-ADPr. ADPr and *iso*-ADPr are depicted as thin gray and orange sticks, respectively. The binding site residues of both structures are labeled (RNF146-*iso*-ADPr numbers in brackets) and possible interactions of *iso*-ADPr with RNF146 are shown as black dashes. **d** Superposition of the TRIP12-ADP WWE domain (slate blue) with the RNF146-*iso*-ADPr WWE domain (green) showing the differences in the binding site of the distal ribose and phosphate groups of *iso*-ADPr. ADP and *iso*-ADPr are rendered as thin cyan and orange sticks respectively. The binding site residues of both structures are labeled (RNF146-iso-ADPr numbers in brackets) and possible interactions of *iso*-ADPr with RNF146 are shown as black dashes.

Unlike RNF146 WWE, the single domain WWE family members HUWE1 and TRIP12 exhibit higher potency for ADPr over *iso*-ADPr in our binding assays (Table 1). The HUWE1-ADPr and TRIP12-ADP crystal structures revealed high conservation of the ligand-binding sites, with most of the ligand interactions being conserved (Fig. 4a, b). The phosphate groups of the two ligands occupy pockets with favorable polar and positively charged groups, interacting with Tyr1658, Asn1669, Thr1672, Asn1674, and Arg1676 in HUWE1 and Tyr809, Asn820, Thr823, Thr825, Arg827 in TRIP12. Despite the terminal ribose having electron density in the HUWE1-ADPr structure (Fig. S9), it points towards the protein surface with no additional directed protein–ligand interactions (Fig. 4a), indicating that the ADP moiety may be the smallest unit that HUWE1 and TRIP12 recognize.

An overlay of the reported RNF146-*iso*-ADPr structure (PDB ID: 3V3L) with the HUWE1-ADPr (Figs. S11b and 4c) and TRIP12-ADP (Figs. S11c and 4d) co-crystal structures revealed two differences involving binding of the distal end of *iso*-ADPr to RNF146. Firstly, Tyr107 that makes a key interaction with the distal ribose oxygen of *iso*-ADPr in RNF146, is replaced by tryptophans in HUWE1 (Trp1619) and TRIP12 (Trp771)—the tryptophans would be incapable of making a similar interaction with *iso*-ADPr. Secondly, the region interacting with the distal phosphate of iso-ADPr in RNF146 contains suitable polar and positively charged residues, while the corresponding region in HUWE1 and TRIP12 contain some acidic residues including two aspartic acids (1621–1622 in HUWE1 and 773–774 in TRIP12) (Fig. 4c and d), making it less positively charged, and therefore less favorable for potential interactions with the distal phosphate moiety of *iso*-ADPr, explaining the lower *iso*-ADPr potencies observed. Taken together, structural and affinity data suggest that the WWE pocket of HUWE1 and TRIP12 recognize the terminal ADP moiety of PAR, rather than the *iso*-ADPr moiety as described for RNF146.

We observed much weaker binding of TRIP12 WWE to the 11-mer PAR polymers compared to RNF146, HUWE1, and DTX1 WWE proteins (Table 1). This was surprising since TRIP12 WWE is evolutionary closely related to HUWE1 WWE (Fig. 1c). An overlay of the TRIP12-ADP and HUWE1-ADPr structures show a highly conserved ADP binding site, with the substitution of Thr825 in TRIP12 with Asn1674 in HUWE1 being one of the small differences (Fig. S11d). A mutation of Asn1674 to threonine in HUWE1 had no effect on PAR11-mer binding (Fig. S12), indicating that the potency of PAR binding is determined by factors outside of the ligand-binding pocket, possibly including the PAR polymer structure.

### The structures of DTX1 in complex with ADP

**Fig. 5:**
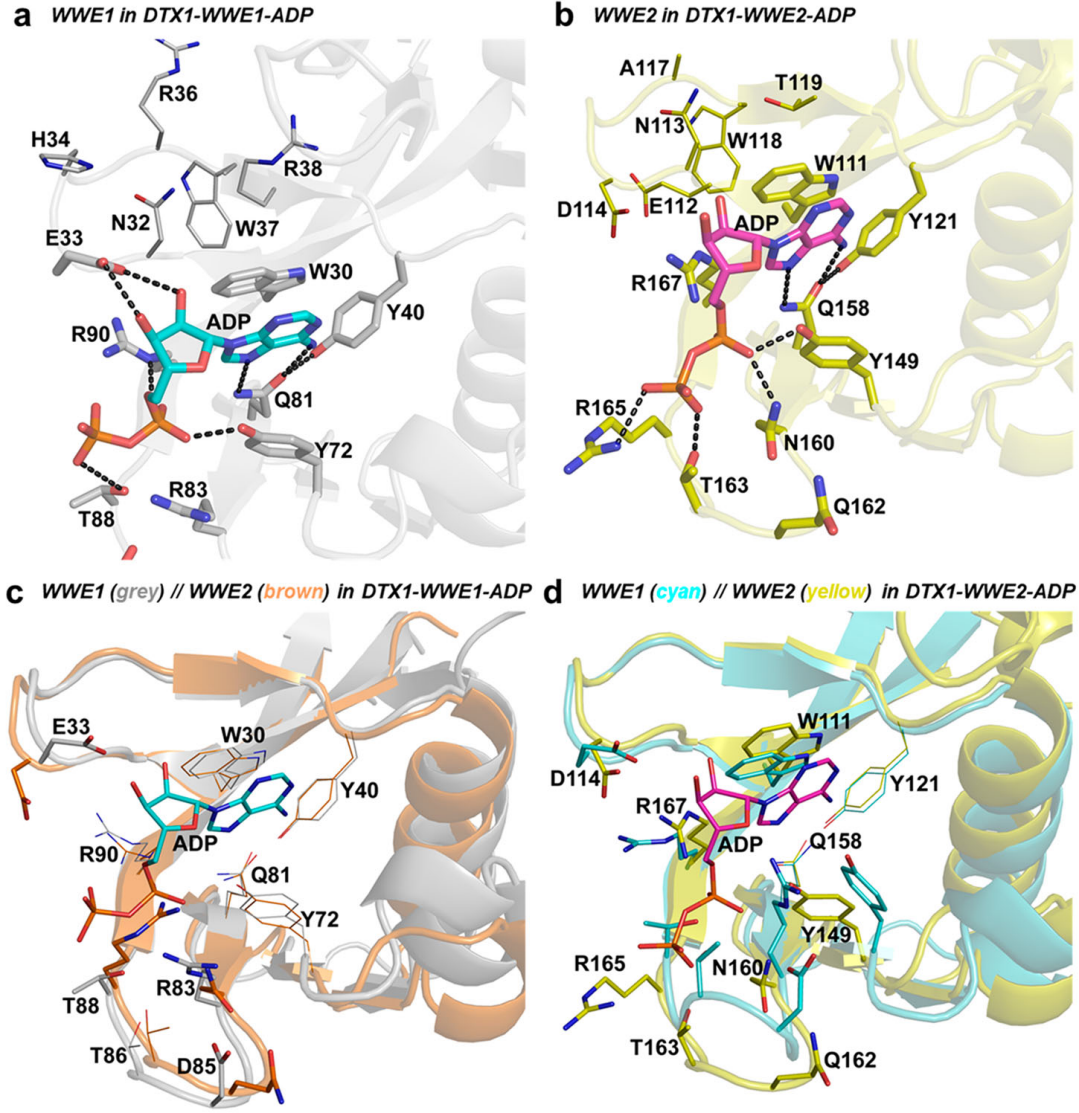
The crystal structures of the DTX1 tandem WWE domains bound to ADP. **a** The binding site of the WWE1 domain in the DTX1-WWE1-ADP structure. ADP is rendered as cyan sticks, the interacting residues are shown as sticks and potential hydrogen bonds are shown as black dashes. Residues depicted as lines correspond to the binding site of the distal phosphate group of *iso*-ADPr as in the RNF146-*iso*-ADPr structure (PDB: 3V3L). **b** The binding site of the WWE2 domain in the DTX1-WWE2-ADP structure. ADP is depicted as magenta sticks and potential hydrogen bonds are shown as black dashes. Similarly, residues depicted as lines correspond to the binding site of the distal phosphate group of *iso*-ADPr as in the RNF146-*iso*-ADPr structure (PDB: 3V3L). **c** A superposition of ADP-bound WWE1 domain (gray) and the unbound WWE2 domain (brown) in the DTX1-WWE1-ADP structure. ADP is shown as thin cyan sticks, conserved residues between the two domains are shown as lines, while residues that are different in type or side chain location are shown as thin sticks. **d** A superposition of ADP-bound WWE2 domain and the unbound WWE1 domain in the DTX1-WWE2-ADP structure. ADP is rendered as thin magenta sticks, residues that are conserved in type and location are shown as lines, while residues that are different in type or side chain location are shown as thin sticks.

The tandem WWE domain proteins DTX1 and DTX2 bind to ADPr and *iso*-ADPr ligands, with varying affinities in the micromolar range, while they bind potently to 11-mer PAR polymers (Table 1). We solved two crystal structures of DTX1 in complex with ADP, with each structure having one ADP molecule bound to either of the two WWE domains (Fig. 5a, b). Our efforts to obtain a DTX1 co-crystal structure with both WWE domains bound to ADP were unsuccessful despite having excess ADP in our crystallization setups. The key ADP-interacting residues in DTX1 WWE1 and WWE2 domains are shown in Figs. 5a and 5b, respectively. In addition to the conserved adenine ring interactions, the ribose and phosphate moieties of ADP interact with polar and charged residues, some of which are conserved in both domains (Fig. S13) and appear to bind in a manner similar to the non-tandem WWE domains of HUWE1 and TRIP12 (Fig. 4a, b).

To gain insight into why ADP ligands were only observed in one WWE domain in our DTX1-ADP structures, we investigated conformational differences by comparing the bound and unbound WWE domains in the same structure (Fig. 5c, d). While the core structural elements appear to be conserved, some loop shifts are observed in both structures, with most differences occurring when ADP is bound to the WWE2 domain as compared to the unbound WWE1 domain in the DTX1-WWE2-ADP structure (Fig. 5d). The observed differences are in the loops that interact with the phosphate groups and may reduce the surface area of the ligand-binding site in the unbound WWE1 domain of the DTX1-WWE2-ADP structure, which therefore might discourage binding in the second site. This would, however, not be the case in the DTX1-WWE1-ADP structure, as only small conformational differences are observed.

Comparing our DTX1-WWE1-ADP and DTX1-WWE2-ADP co-crystal structures with the RNF146-*iso*-ADPr structure revealed that the residues at the corresponding distal ribose-phosphate moeities binding sites in DTX1 WWE domains may not support high affinity binding to *iso*-ADPr (Fig. S14). Specifically, neither Trp30 nor Trp111 would allow for a direct hydrogen bond to the distal ribose oxygen, while residues in the loop regions (30–38 in WWE1 and 111–122 in WWE2) would not contribute to high affinity binding to the distal phosphate group. Our biophysical data, however, supports DTX1 preference for *iso*-ADPr over ADPr (Table 1), which may indicate a mechanism where it recognizes PAR *via* both *iso*-ADPr and ADP moieties.

### The model of the DTX2 WWE domain

Our DTX2 WWE protein showed binding to the 11-mer PAR polymers, as well as ADPr (Table 1 and Fig. 2a), however, our efforts to crystalize the protein alone or in complex with ADP and ADPr were unsuccessful. We therefore examined the AlphaFold-predicted structure to gain insights into the fold and the binding sites of the DTX2 WWE domains. As expected, the predicted DTX2 WWE domain has a similar fold as observed in the DTX1-ADP co-crystal structures, with the binding pockets having all conserved residues in place (Fig. S15). Based on this, we expect the PAR-building blocks ADP and ADPr to bind in a manner similar to ADP in our DTX1-ADP co-crystal structures (Fig. 5), suggesting that DTX2 may also recognize PAR via the ADP moiety.

### The structures of the WWE domain proteins with ATP and analogs thereof

In this study, we developed an NMR-based displacement assay that can be used to screen for WWE domain binders using ATP and two fluorinated analogs, with the three ligands having varied binding affinities in the micromolar range to our WWE proteins (Table 1). To visualize the interactions of ATP with the WWE domains, we solved the crystal structures of TRIP12 and DTX1 in complex with ATP (Fig. S16). The TRIP12-ATP structure shows ATP binding in a similar manner to ADP in the TRIP12-ADP structure, with the only difference being the position of the Arg807 sidechain, which interacts with the terminal phosphate group of ATP as opposed to interacting with the ribose oxygen in the TRIP12-ADP structure (Fig. S16a, b). The DTX1-ATP structure has one ATP molecule in the WWE1 domain while WWE2 is unbound, a trend observed with our DTX1-ADP structures. The ATP molecule binds the same way as ADP in the DTX1-ADP structures, with the terminal phosphate group of ATP having no interactions with the protein residues (Fig. S16c, d). Taken together, the two ATP co-crystal structures provide a detailed characterization of TRIP12 and DTX1 interaction with ATP and show that the interaction with the ADP moiety of ATP is preserved. The reported structure of the mouse RNF146 WWE in complex with ATP^49^ shows very similar interactions of the ADP moiety with the conserved binding site residues (Fig. S17a). A superposition of this structure with our ATP bound structures (Fig. S17b, c) highlights the conservation of ATP binding to the proteins, with the exception of the terminal phosphate group, which adopts different conformations based on the residues in the interacting loop.

Based on the high binding affinity of the HUWE1 WWE domain to 2’F-ATP (**2**) (15 µM, ~2.5-fold higher than for ADPr; Table 1), we solved the co-crystal structure with 2’F-ATP revealing a different binding mode compared to ADPr (Fig. S18a, b). The fluorine group of 2’F-ATP (**2**) overlays with the pyrazole nitrogen of the adenine backbone in the ADPr binding site. The ligand mainly interacts with a network of water molecules and a hydrogen bond of a ribose oxygen to Gln1667, as well as *π-π* interactions with the conserved Trp1619 aromatic sidechain.

### Application of the toolbox for WWE domain ligand discovery

**Fig. 6:**
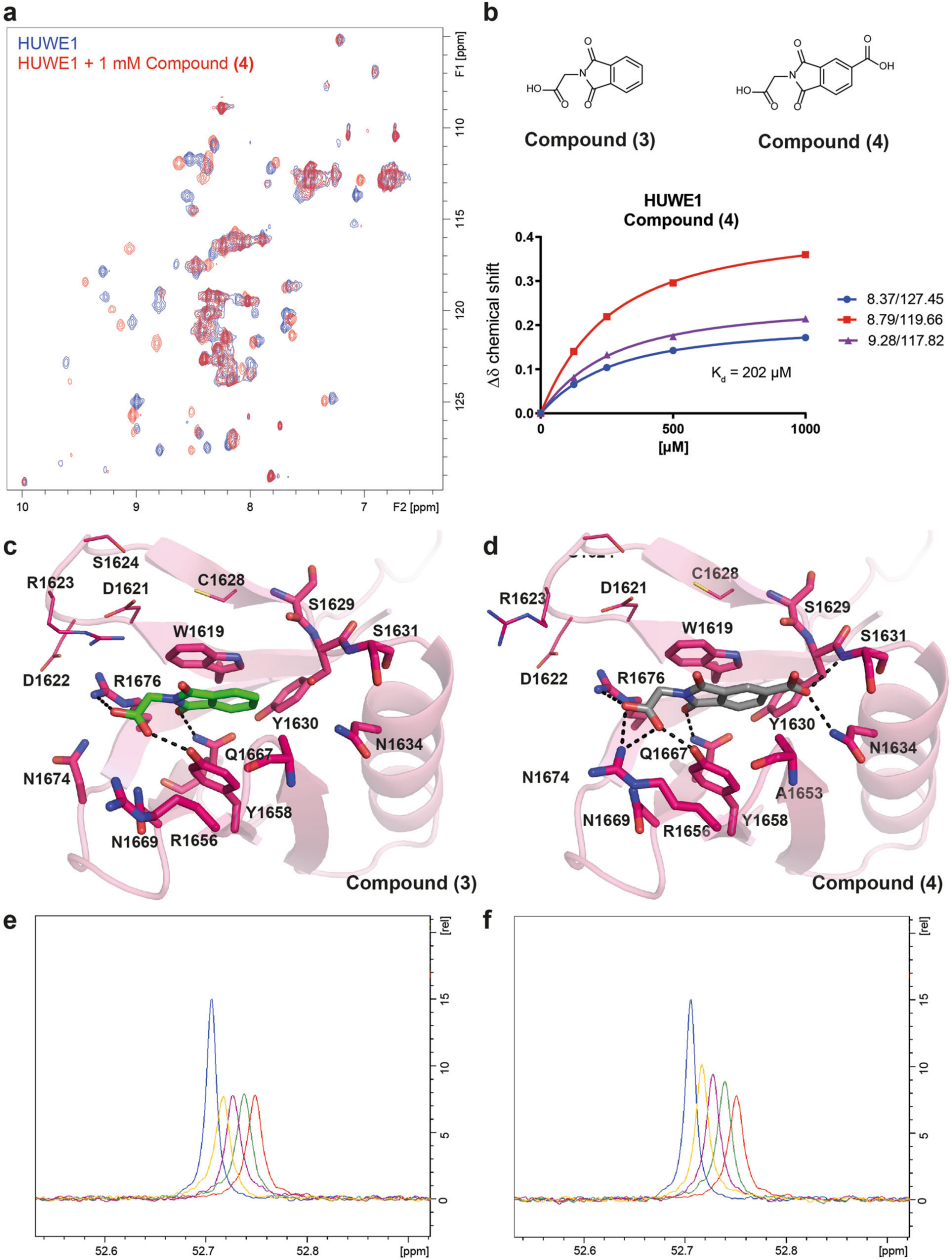
Two small molecules bound to the HUWE1 WWE domain. **a** ^15^N-HSQC spectra of HUWE1 in blue lines overlaid with protein in the presence of 1 mM compound (**4**) in red lines. **b** Chemical structures of (**3**) and (**4**). Dissociation constant curve of 125 µM to 1000 µM compound (**4**) titrated to HUWE1 WWE domain protein at 100 µM. NMR *K*_d_ values originate from distinct samples (*n* = 1) measured for each concentration, mean *K*_d_s are obtained from curves of selected cross peaks ± standard deviations. **c**, **d** The binding site of the HUWE1 WWE domain. **c** Compound (**3**) is rendered as green sticks and **d** compound (**4**) is rendered as gray sticks, the interacting residues are shown as sticks and potential hydrogen bonds are shown as black dashes. **e**, **f** ^19^F-Displacement assay with HUWE1 WWE domain and **e** compound (**3**) and **f** compound (**4**). The F-ATP reporter is in blue, the F-ATP reporter with HUWE1 WWE domain protein is in red and the titration of **e** compound (**3**) or **f** compound (**4**) at 250 mM (green), 500 mM (magenta) 1000 mM (yellow). ^19^F signals are displayed at an offset of 0.01 ppm to enhance clarity.

Following the successful development of hit finding infrastructure, we set out to evaluate the small molecule ligandability of the WWE domains. In our case study, we screened 8000 fragments of the Boehringer Ingelheim (BI) library against the HUWE1 WWE domain using HSQC NMR as the primary screening method. Intriguingly, we identified two phthalimide-based scaffolds, N-(Carboxymethyl)-phthalimide (**3**) and its respective 4-carboxy derivative (**4**). Both molecules induced chemical shift perturbations in the HSQC spectrum and were then subjected to HSQC titrations, yielding a *K*_d_ of 1763 µM for compound (**3**) and a stronger affinity for compound (**4**) with a *K*_d_ of 202 µM (Fig. 6a, b), the latter one was one of the most potent fragments identified in the screen. To elucidate the binding mode and rationalize the structure-affinity relationship, we generated crystal structures of the compounds (**3**) and (**4**) in complex with the HUWE1-WWE domain (Fig. 6c, d, Table 3). Both structures exhibited unambiguous electron density for the fragment molecules (Fig. S9), confirming a conserved binding mode in which one of the phthalimide carbonyl oxygen atoms forms a direct hydrogen bond with the nitrogen of the Gln1667 (2.8 Å and 2.7 Å, respectively). The aromatic system stacked parallel to Trp1619 with a plane distance of 4.1 Å and with a weak CH-O interaction to the Gln1667 oxygen (3.5 Å and 3.5 Å, respectively). The methyl-carboxylic acid formed a complex hydrogen network through direct hydrogen bonds to Tyr1658, Asn1669, and Arg1676, thereby mimicking the phosphate-1 observed in the ADPr complex structure. The second carboxyl group of compound (**4**) formed two additional hydrogen bonds, one with the side chain nitrogen of Asn1634 (2.9 Å) and another with the backbone nitrogen of Ser1631 (3.2 Å). Overall, the binding mode of the phthalimide scaffold overlays with the adenine-backbone of the natural substrate-derived ADPr and the 2’F-ATP (**2**) molecule, suggesting competitive behavior. We tested the utility of the discovered fragments using the ^19^F-NMR-based displacement assay to investigate whether compound (**3**) and (**4**) led to the displacement of reporter 2F-ATP (**1**). In line with the NMR-based *K*_d_ measurements for the weaker compound (**3**), no displacement could be observed at concentrations up to 1 mM (Fig. 6e), whereas a clear dose-dependent displacement for the more potent compound (**4**) was observed (Fig. 6f).

## Discussion

E3 ligases have gained increased attention over the past 20 years due to their application as tools for degradation of protein targets. Only a small fraction (~2.4%) of the  more than 600 E3 ligases have been utilized for targeted protein degradation, with von Hippel-Lindau (VHL) and cereblon (CRBN) being the most harnessed E3 ligases to date. In this study, we set out to characterize physiological ligands binding to the WWE domains of all WWE-containing subfamily of E3 ligases that include RNF146, TRIP12, HUWE1, and DTX 1, 2, and 4. We expressed and purified WWE proteins for all family members except DTX4 and assessed their binding to PAR and its derived ligands using NMR and FP. Importantly, we generated the first crystal structures of human HUWE1, TRIP12, and DTX1 WWE domains in the presence of PAR-derived ligands as well as ATP analogs, increasing the structural understanding of PAR and nucleotide binding interactions.

Our study shows that the WWE domain and PAR binding sites are conserved, but RNF146 specifically stands out as a family member, exhibiting higher binding affinities for PAR chains and the *iso*-ADPr subunit. Based on the structural and biophysical data, we hypothesize that other family members preferentially recognize the ADPr moiety of PAR and are therefore likely to bind both mono- and poly-ADP-ribosylated substrates in the cells. The weaker interactions of PAR with the other WWE family members are likely to be more transient and may be complemented by additional interactions of the E3 ligase with the target proteins. For HUWE1 it has already been shown that an intrinsically disordered region is important for histone H1 ubiquitination of a ΔIDR1 variant^13^.

Finally, we ran a pilot experiment to identify small molecule binders of the HUWE1 WWE domain by employing the assays and resources developed in this study, specifically using NMR to screen an 8000-fragment library and structural studies to visualize the interactions of the hit molecules with the protein. We identified two HUWE1 WWE binders and the structural data revealed that the hit fragments occupy the PAR binding site and exhibit early signs of a structure-activity relationship. The stronger binding compound (**4**) showed displacement of the ^19^F reporter (**1**) indicating that the ^19^F NMR displacement assay could serve as a driving assay for optimization of compounds exhibiting *K*_d_ values < 200 µM. The observations made in this experimental study are encouraging, both in terms of assay suitability and general ligandability of HUWE1 and the other E3 ligase WWE domains.

In summary, as an initial step in catalyzing the development of chemical tools and probes for the E3 ligase WWE domain class, we have developed a toolbox for hit finding and characterization. The assays presented in this study have the potential to guide hit discovery research from fragment-based approaches of hits starting in the mM range (HSQC *K*_d_) to later optimization phases (^19^F displacement and PAR-FAM assays), with the crystallization protocols enabling structure-guided characterization and optimization. We believe that the data and tools presented in this study will support the generation of small molecule binders and chemical probes for the WWE domain-containing E3 ligases.

## Methods

### Sequence alignment and phylogenetic tree of the E3 ligase WWE domains

All E3 ligase WWE protein sequences were obtained from UniProt. Multiple sequence alignment of the WWE domains was generated using Clustal Omega^51^ and manually optimized based on the available crystal structures to generate a structure-based alignment, then annotated using the ESPript 3.0 server^52^ to highlight conservation and secondary elements relative to the HUWE1 WWE apo structure (PDB ID: 6MIW). To generate phylogenetic trees, the NGPhylogeny suite^53–57^ was used to calculate phylogenetic tree strings that were used as input into the interactive tree of life (iTOL)^58^ to render the trees.

### Protein preparation

#### WWE domain gene cloning, protein expression, and purification

The genes of the WWE domains from HUWE1, TRIP12, RNF146, DTX1, DTX2, and DTX4 human E3 ligases (Table S1) were cloned into an in-house *E. coli* expression vector pET28-MHL, yielding expression constructs with an N-terminal His_6_-tag followed by a TEV cleavage site. All proteins were expressed overnight at 16 °C in *E. coli* BL21 (DE3) pRARE2 cells. The cells were harvested by centrifugation and lysed by sonication, followed by centrifugation to collect the supernatant (cell-free extract). The HUWE1-WWE (N-terminal His-SUMO-tag) construct for crystallization and NMR experiments was kindly provided by Tim Clausen (IMP, Vienna, Austria) and the protein was expressed overnight at 20 °C in *E. coli* BL21 (DE3) cells. For protein-observed ^15^N-HSQC NMR applications all WWE domains were grown in M9 minimal medium supplemented with ^15^NH_4_Cl (0.5 g l^−1^).

Protein purification was performed by Nickel immobilized metal affinity chromatography. Briefly, the supernatant was incubated with Nickel affinity resin in an open column, following which the unbound proteins were removed and the resin washed three times with a low imidazole buffer before elution with a buffer containing 250 mM imidazole. The eluted WWE proteins were subjected to polyhistidine purification tag removal by cleavage with the TEV protease overnight, after which the protein samples were applied to Nickel resin and the unbound (cleaved) proteins collected. For the HUWE1-WWE construct for NMR, the His-SUMO-tag was cleaved with SENP2 protease overnight.

The collected proteins were concentrated and loaded onto the HiLoad^TM^ 16/60 Superdex^TM^ 75 gel filtration column (on an ÄKTA Pure chromatography system (GE Healthcare)) running in the final protein buffer for each protein as described in Table 1. Protein fractions containing pure WWE domain proteins as confirmed by SDS-PAGE were pooled and concentrated using a 3 kDa cutoff protein spin concentrator (Millipore). The final protein concentration was determined using a Nanodrop (Thermo Scientific), with the protein extinction coefficient computed from the respective amino acid sequence using Expasy ProtParam^59^.

### Fluorescence polarization-based 11-mer PAR binding assays

PAR was synthesized enzymatically as described previosuly^60^. Briefly, PARP5a catalytic domain (0.1 mg mL^−1^), histones (1 mg mL^−1^) and NAD^+^ (20 mM) were incubated in 100 mM Tris pH 8.0, 10 mM MgCl_2_, 1 mM DTT for 1 h at 30 °C. The proteins were precipitated with 10% (v/v) trichloroacetic acid, and the PAR cleaved from the proteins with 0.5 M KOH, 50 mM EDTA for 2 h at 60 °C. 11-mer PAR was purified from the PAR mixture with a DNAPac-PA100 anion exchange column attached to an Agilent Infinity 1260 HPLC. The 11-mer PAR was concentrated, desalted, then enzymatically labeled with dATP-FAM at its 2’-end as described^61^. The FAM-labeled 11-mer PAR was then purified from the labeling reaction with ion-pairing reverse-phase HPLC as described^62^.

FP assays were carried out to assess the binding of all purified WWE domain proteins to a FAM-labeled 11-mer poly-ADPr (FAM-PAR) oligonucleotide. All experiments were performed in a total assay volume of 10 µL per well in 384-well black polypropylene PCR plates (PCR-384-BK, Axygen, Tewksbury, MA). For direct binding of FAM-PAR to the WWE domain, varying concentrations of the protein were incubated at room temperature for 30 min with 25 nM FAM-PAR in 20 mM Tris-HCl, pH 7.5, buffer containing 150 mM NaCl and 0.01% (v/v) Triton X-100. A blank reaction containing FAM-PAR in assay buffer was included in all experiments. FP was measured at room temperature using a BioTek Synergy 4 (BioTek, Winooski, VT) with excitation and emission wavelengths of 485 nm and 528 nm, respectively. All experiments were performed in triplicate with three independent repeats. The FP values were blank-subtracted and the change in FP (mP) was plotted as a function of the WWE domain protein concentration. The concentration of protein corresponding to the half-maximum FP signal (*K*_d_) was calculated using nonlinear least-squares regression to a single-site binding model (GraphPad Prism 9.5, GraphPad Software, Boston, Massachusetts USA).

To establish the specificity of the FP assay, a FP displacement was performed on the HUWE1 WWE protein. A mixture containing the HUWE1 WWE domain protein and 25 nM FAM-PAR in 20 mM Tris-HCl, pH 7.5, buffer containing 150 mM NaCl and 0.01% (v/v) Triton X-100 was pre-incubated for 15 min before varying concentrations of unlabeled 11-mer poly ADPr (11-PAR) were added and incubation continued for a further 30 min at room temperature. FP was measured in 10 μL reaction volumes as described above. The FP values were determined, and the *K* displacement (*K*_disp_) value (the concentration required for 50% displacement of the labeled PAR oligo) were calculated using nonlinear least-squares regression analysis to a four-parameter concentration-response curve model (GraphPad Prism 9.5, GraphPad Software, Boston, Massachusetts USA).

### Synthesis of *iso*-ADPr

*Iso*-ADPr was synthesized according to a previously published protocol with the following differences: a 30 mL in vitro PARylation reaction was used with a three-fold increased concentration of reactants^63^.

### ^19^F-displacement assay

NMR experiments were performed on a Bruker AVII 600 MHz spectrometer equipped with a 5 mm z-gradient QCI cryogenic probe (^15^N/^13^C/^19^F/^1^H) and a SampleJet™ sample changer. As a reference, 100 µM F-ATP (in H_2_O) in buffer (20 mM Tris, 100 mM NaCl, 5% DMSO, 2% D_2_O) was measured. Studied E3 ligases were added to the sample at 0.5 µM for DTX1, DTX2, TRIP12, and HUWE1 and at 2 µM for RNF146. Competitor (ATP in H_2_O) was titrated at concentrations of 250 µM, 500 µM and 1000 µM with the NMR sample.

### ^15^N-HSQC-NMR

NMR experiments were performed on a Bruker AVII 600 or 700 MHz spectrometer equipped with a 5 mm z-gradient QCI cryogenic probe (^15^N/^13^C/^1^H) and a SampleJet™ sample changer. For *K*_d_ determinations by NMR, 70 µM uniformly ^15^N-labeled WWE domain proteins of HUWE, TRIP12, RNF146, DTX1, and DTX2 were mixed with two-fold increasing concentrations of ligand (substrate-derived nucleotides and ATP analogs) from 31.25 µM to 2 mM at a constant DMSO concentration of 1% (v/v) ^15^N-HSQC NMR spectra were recorded, and chemical shift perturbations were analyzed with Topspin 3.6 software from Bruker. Titration curves were calculated as previously described^64^.

### Protein crystallization

Purified TRIP12 protein was co-crystallized with ADP and ATP nucleotides using the sitting drop vapor-diffusion method. The protein at 20-30 mg mL^−1^ (2.2–3.3 mM) concentration in the final protein buffer (20 mM Tris-HCl pH 7.5, 150 mM NaCl, 1 mM TCEP) was mixed with a 10 fold molar excess (22–33 mM) ADP or ATP and incubated at room temperature for 15 min prior to crystallization set-up. Crystallization was carried out via screening using Morpheus® (Molecular Dimensions) and Redwings® in-house screening kits, with equal volumes of the protein-nucleotide complex and the precipitant solution in 1 μL drops over 90 μL reservoir solution, using the original INTELLI-PLATE 96-2 (ART Robbins Instruments) sitting drop vapor diffusion plates. Crystals were observed within 72 h at 18 °C in precipitant solutions containing: (i) 12.5% MPD, 12.5% PEG1000, 12.5% PEG3350, 0.1 M Sodium Hepes/MOPS pH 7.5, 0.09 M Sodium nitrate, 0.09 M Sodium phosphate dibasic, 0.09 M Ammonium sulfate for TRIP12-ADP, and (ii) 25% P3350, 0.2 M MgCl, 0.1 M Tris-HCl, pH 8.5 for TRIP12-ATP crystals. Crystals were cryoprotected by briefly soaking in solutions containing crystallization mother liquor supplemented with 10% ethylene glycol where necessary and 1 mM respective nucleotide, before freezing in liquid nitrogen.

The HUWE1 and DTX1 WWE ligand co-crystal structures were generated by soaking apo crystals with the respective ligands. HUWE1-WWE domain crystals were obtained by mixing 200 nL protein solution (88.0 mg mL^−1^, 20 mM Tris pH 8.0, 100 mM NaCl) with 200 nL reservoir (0.1 M NaOAc pH 4.83, 2.9 M NaCl) on SWISSCI MRC 2 plates at 4 °C. DTX1-WWE domain crystals were obtained by mixing 300 nL protein solution (7.44 mg mL^−1^, 20 mM Tris pH 7.5, 150 mM NaCl, 5% glycerol, 1 mM TCEP) with 150 nL reservoir (0.1 M Tris pH 8.0, 25% v/v PEG MME 350) on SWISSCI MRC 2 plates at 20 °C. For protein–ligand complex structures of HUWE1 WWE and DTX1, the solvent of 1 µl of nucleotide solution (100 mM in H_2_O) and small molecule solution (50 mM in DMSO d_6_) was evaporated and the ligands were re-dissolved in soaking buffer (0.1 M NaOAc pH 4.83, 2.9 M NaCl, 25% ethylene glycol). Fully-grown crystals were transferred in the soaking buffer with ligands and soaked for 24 h. Crystals were flash-frozen in liquid nitrogen.

### Diffraction data collection, structure determination. and refinement

Diffraction data were collected on beamline 24-ID-C at the Advanced Photon Source in the Argonne National Laboratory and on beamline X10SA of the Swiss Light Source (Paul Scherrer Institute, Switzerland). The diffraction data were processed with HKL3000^65^ and XDS^66^ and structures were solved by molecular replacement in Phaser^67^ using the HUWE1 WWE domain crystal structure (PDB ID: 6MIW) as a starting model. The models were refined by alternating cycles of manual rebuilding in Coot^68^ and refinement with Refmac^69^ within the CCP4 crystallography suite^70^. The structures were validated using the Molprobity server^71^ and analyzed using UCSF Chimera^72^, and the molecular graphics images were rendered using PyMOL (The PyMOL Molecular Graphics System, Version 4.6 Schrödinger, LLC).

### HUWE1 small molecule identification by NMR

The proprietary Boehringer Ingelheim fragment library (8000 fragments) was screened in mixtures of ten compounds with each compound at 20-fold excess against 50 µM HUWE1 WWE in 25 mM Bis Tris, 150 mM NaCl, pH 6.5, on an Avance III 700 MHz spectrometer equipped with a 5 mm z-gradient QCI cryogenic probe (^15^N/^13^C/^1^H) and a SampleJet™ sample changer. ^15^N-HSQC spectra were recorded and compound hits in the mixtures were identified by comparison with ^15^N-HSQC reference spectra of 50 µM HUWE1 WWE with DMSO. Compound hit confirmation was carried out in single compound samples using the same conditions and parameters as for compound mixtures.

### Compound availability

Compound (**1**) and (**2**) were purchased at Jena Bioscience (NU-145S and NU-151S, respectively). compound (**3**) and compound (**4**) are available via Sigma-Aldrich (P40506) and Chembridge (# 5140421), respectively.

### Statistics and reproducibility

All statistics are described in the “Methods” and figure legends.

### Reporting summary

Further information on research design is available in the Nature Portfolio Reporting Summary linked to this article.

### Supplementary information

Supplemental Information

Description of Additional Supplementary Files

Supplementary Data

Reporting summary

## Data Availability

The model coordinates are deposited in the RCSB PDB under PDB IDs: 7UW7 (TRIP12-ADP), 8TRE (TRIP12-ATP), 8R7O (HUWE-2’F-ATP (2)), 8R6A (DTX1-ADP-WWE1), 8R6B (DTX1-ADP-WWE2), 8R5N (DTX1-ATP), 8RE1 (HUWE1-ADPr) 8RD0 (HUWE1-Compound (3)) and 8RD1 (HUWE1- Compound (4)). The source data behind the graphs in the paper can be found in the Supplementary Data files.
