# Supplementary material for: A ligand discovery toolbox for the WWE domain family of human E3 ligases
Source: Commun Biol. 2024 Jul 24;7:901. doi: 10.1038/s42003-024-06584-w (PMC11269756; doi:10.1038/s42003-024-06584-w)
Supplement: Supplementary file 1 — Supplemental Information [file 42003_2024_6584_MOESM1_ESM.pdf]

Table S1: Construct boundaries for the WWE domain proteins purified in this study and their respective protein buffers.

Protein	UniprotKB ID	WWE residues cloned	Protein buffer
DTX1	Q86Y01-1<br>(Isoform 1, 620 aa*)	21-184	20 mM Tris-HCl pH 7.5, 150 mM NaCl, 5% Glycerol, 1 mM TCEP
DTX2	Q86UW9-1<br>(Isoform 1, 622 aa)	3-189<br>(3-187)	20 mM Tris-HCl pH 8.0, 250 mM NaCl, 5% Glycerol, 1 mM TCEP
TRIP12	Q14669-2<br>(Isoform 2, 2025 aa)	761-839<br>(759-847)	20 mM Tris-HCl pH 7.5, 150 mM NaCl, 1 mM TCEP
RNF146	Q9NTX7-1<br>(Isoform 1, 359 aa)	100-184	20 mM Tris-HCl pH 7.5, 150 mM NaCl, 1 mM TCEP
HUWE1	Q7Z6Z7-1<br>(Isoform 1, 4374 aa)	1611-1700	20 mM Tris-HCl pH 7.5, 150 mM NaCl, 1 mM TCEP (FP assays)<br>10 mM HEPES pH 7.5, 150 mM NaCl; 1 mM TCEP (NMR and crystallization)

*aa: amino acids

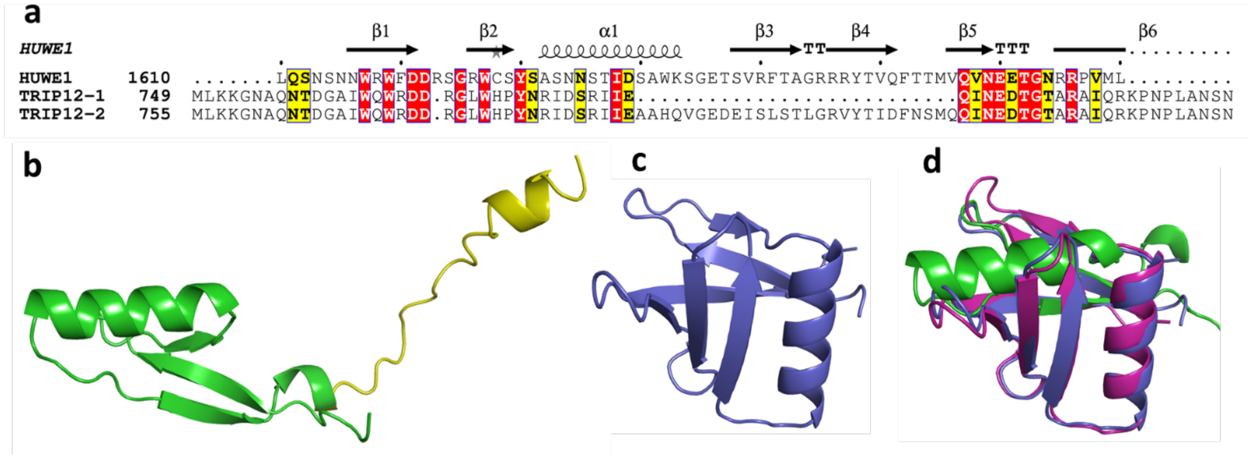

Figure S1. The 28 amino acids that are missing in isoform 1 of the TRIP12 WWE domain. (a) Sequence alignment of the WWE domains of TRIP12 isoforms 1 and 2 with the HUWE1 WWE domain sequence. The secondary structure elements are annotated based on the HUWE1 WWE apo structure (PDB ID: 6MIW). Residues that form β-strands 3 and 4 are missing in isoform 1 of the TRIP12 WWE domain sequence. **(b)** AlphaFold predicted structure of residues 749-836 that are annotated to form the WWE domain in the isoform 1 TRIP12 sequence. **(c)** AlphaFold predicted structure of TRIP12 isoform 2 WWE domain**. (d)** An overlay of the HUWE1 WWE apo structure (PDB ID: 6MIW) and the AlphaFold predicted structures of the isoform 1 (green) and isoform 2 (slate blue) TRIP12 WWE domains. Due to the missing 28-aa segment, the isoform 1 TRIP12 WWE structural fold is altered. In addition, the segment rendered in yellow in the isoform 1 TRIP12 WWE predicted structure (panel A) is not part of the WWE domain despite being annotated so.

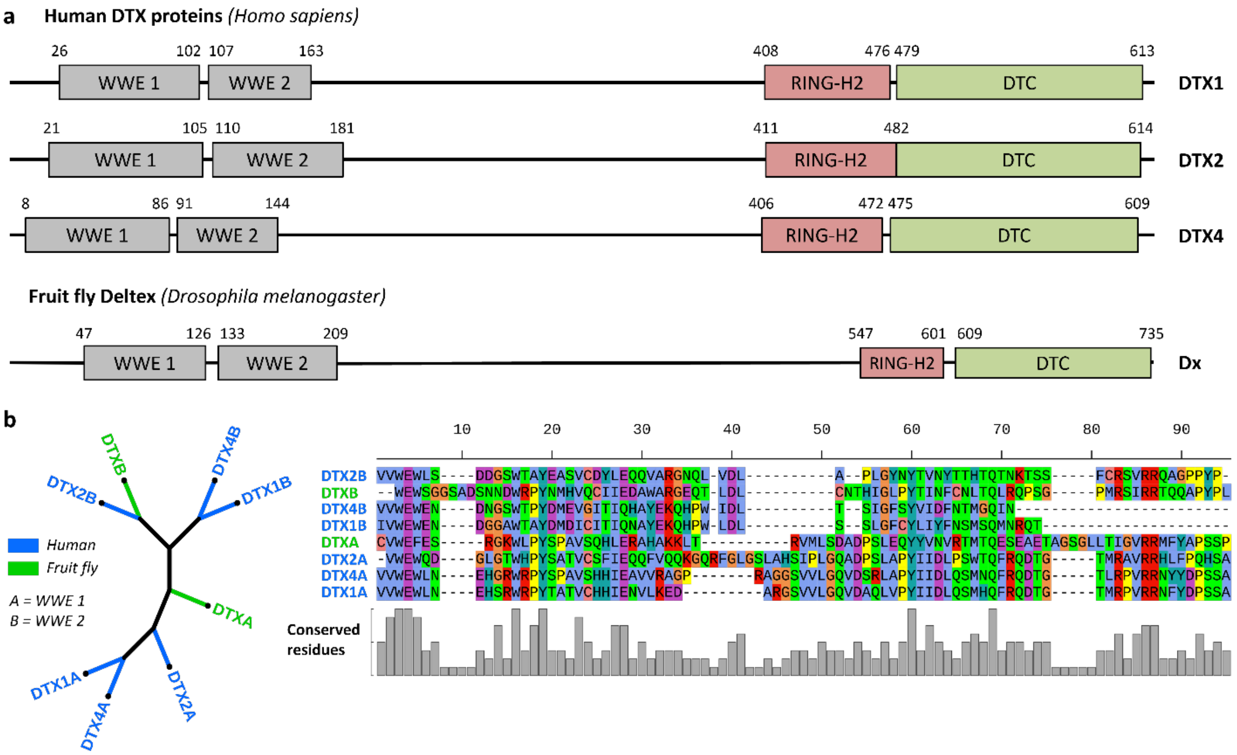

Figure S2: Domain architecture of Deltex E3 ligases containing a WWE domain. (a) Domain organization of human and fruit fly DTX E3 ligases with cutoffs defined by the NCBI conserved domains database. Domains are drawn to scale and are defined as follows: WWE1/2 = first/second WWE domain, RING-H2 = Really Interesting New Gene finger domain, DTC = Deltex C-terminal domain. (**b)** Phylogenetic tree and complete sequence alignment of human DTX1, DTX2 and DTX4 WWE domains with the *Drosophila* Deltex WWE domain. The residues have been re-numbered starting from one and the phylogenetic tree was constructed without considering branch lengths.

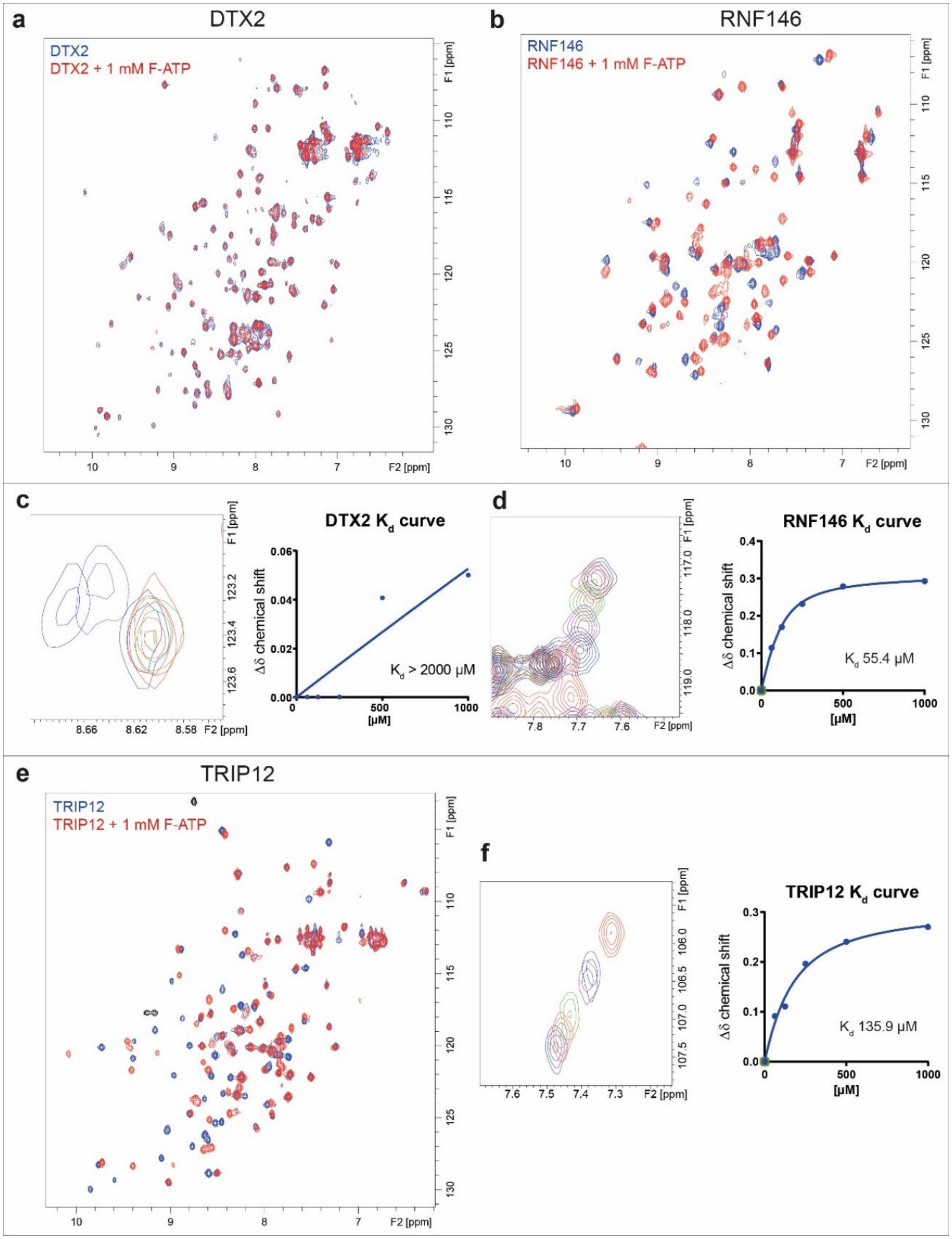

Figure S3: ^{15}N-HSQC NMR based binding studies of ATP-analogues. (a,b,e) ^{15}N-HSQC spectra of **(a)** DTX2, **(b)** RNF146, and **(e)** TRIP12 overlaid with protein in the presence of 1 mM 2F-ATP **(1). (c, d, f)** ^{15}N-HSQC NMR K_d titration assay and zoom-in on a peak upon a twofold titration of 2F-ATP **(1)** to **(c)** DTX2, **(d)** RNF146, and **(f)** TRIP12 using a concentration range of 62.5 μM to 2 mM. NMR K_d values originate from distinct samples (n=1) measured for each concentration, mean Kds obtained from curves of selected cross peaks ± standard deviations. For the titration curve the ligand concentrations are plotted on the x-axis and the Δδ chemical shifts on the y-axis.

a

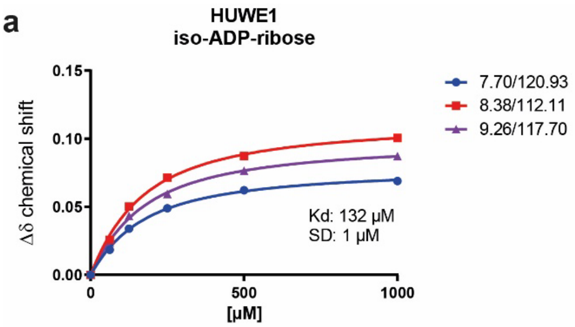

	7.70/120.93	8.38/112.11	9.26/117.70
Best-fit values			
Ptot	= 70.00	= 70.00	= 70.00
Kd	132.0	130.2	133.7
Bmax	0.07953	0.1143	0.09943

b

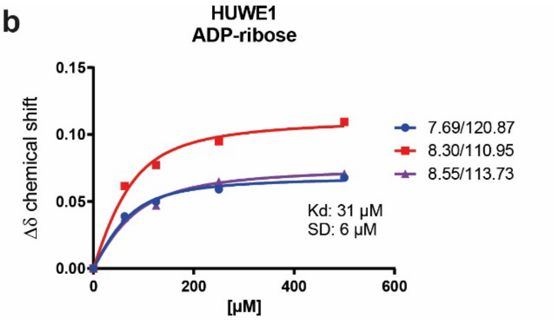

	7.69/120.87	8.30/110.95	8.55/113.73
Best-fit values			
Ptot	= 70.00	= 70.00	= 70.00
Kd	24.19	29.11	39.21
Bmax	0.06932	0.1134	0.07693

c

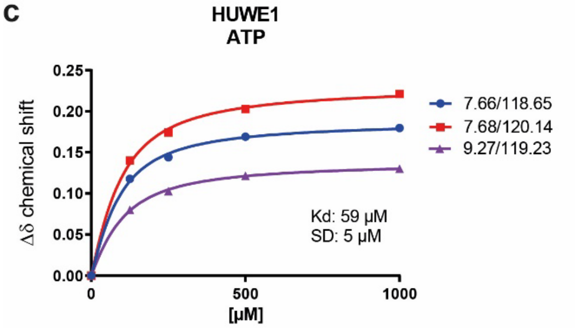

	7.66/118.65	7.68/120.14	9.27/119.23
Best-fit values			
Ptot	= 70.0	= 70.0	= 70.0
Kd	52.9	58.6	64.8
Bmax	0.189	0.232	0.139

d

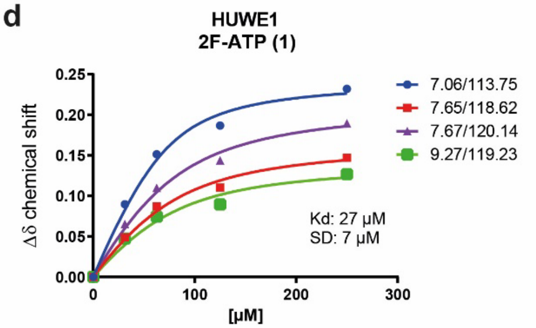

	7.06/113.75	7.65/118.62	7.67/120.14	9.27/119.23
Best-fit values				
Ptot	= 70.0	= 70.0	= 70.0	= 70.0
Kd	14.6	30.6	30.5	30.6
Bmax	0.244	0.167	0.216	0.142

e

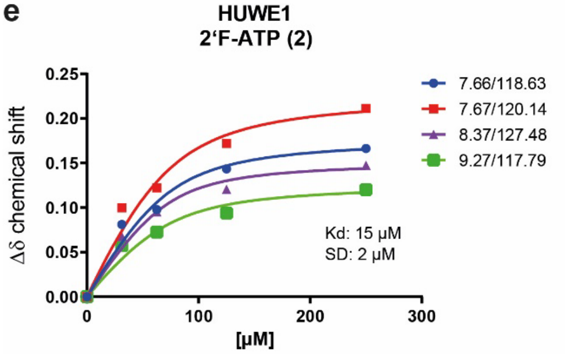

	7.66/118.63	7.67/120.14	8.37/127.48	9.27/117.79
Best-fit values				
Ptot	= 70.0	= 70.0	= 70.0	= 70.0
Kd	14.4	17.5	11.4	15.8
Bmax	0.178	0.227	0.153	0.127

f

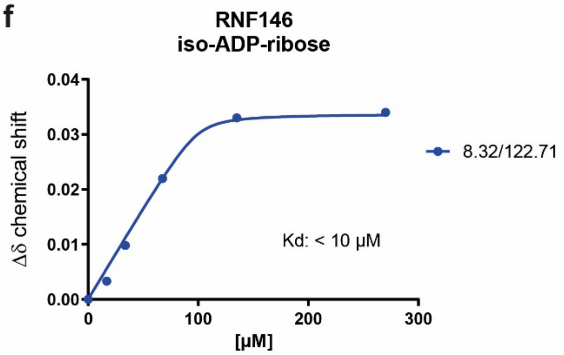

	8.32/122.71
Best-fit values	
Ptot	= 100
Kd	1.35
Bmax	0.0338

Figure S4: ^{15}N-HSQC-NMR titration curves of HUWE1 and RNF146. (a-f) For the titration curve the ligand concentrations are plotted on the x-axis and the Δδ chemical shifts on the y-axis. The maximal plotted concentrations are up to compound saturation. The Δδ chemical shifts of one to four different peaks are followed of a single titration experiment. The position of the individual peak on the ^{1}H- and ^{15}N-scale of a ^{1}H-^{15}N-HSQC spectrum is labeled in ppms in the legend. Below the titration curve is a table with the position of the peaks in the column headers and the protein concentration in the experiment (Ptot), the dissociation constant (K_d) in µM and the maximum specific binding (Bmax) of each of the analyzed peaks in the rows. The titration curve of **(a)** *iso*-ADPr, **(b)** ADPr, **(c)** ATP, **(d)** 2F-ATP **(1)**, **(e)** 2'F-ATP **(2)** to HUWE1 and **(f)** *iso*-ADPr to RNF146 is plotted. .NMR K_d values originate from distinct samples (n=1) measured for each concentration, mean Kds obtained from curves of selected cross peaks ± standard deviations

a

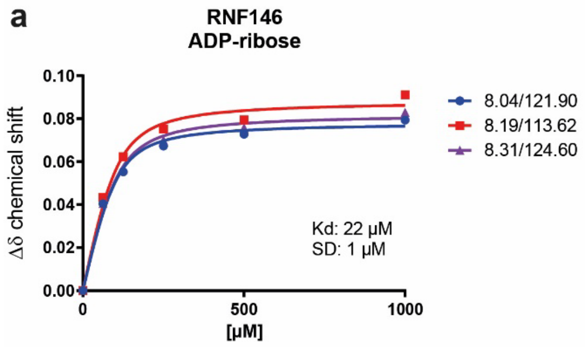

	8.04/121.90	8.19/113.62	8.31/124.60
Best-fit values			
Ptot	= 100	= 100	= 100
Kd	19.6	22.0	23.1
Bmax	0.0782	0.0883	0.0822

b

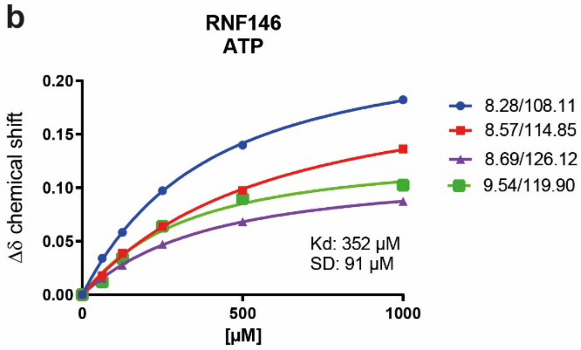

	8.28/108.11	8.57/114.85	8.69/126.12	9.54/119.90
Best-fit values				
Ptot	= 100.0	= 100.0	= 100.0	= 100.0
Kd	322.0	504.2	323.3	259.5
Bmax	0.2447	0.2088	0.1179	0.1356

c

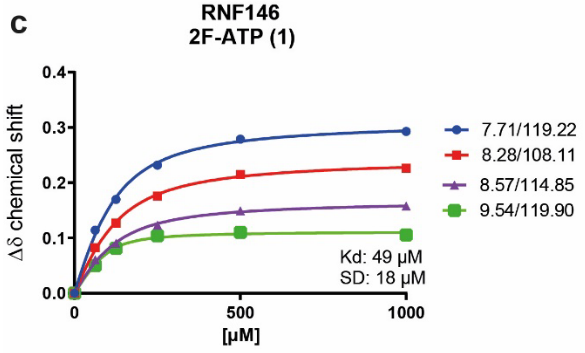

	7.71/119.22	8.28/108.11	8.57/114.85	9.54/119.90
Best-fit values				
Ptot	= 100	= 100	= 100	= 100
Kd	55.4	64.8	58.9	18.4
Bmax	0.312	0.244	0.168	0.112

d

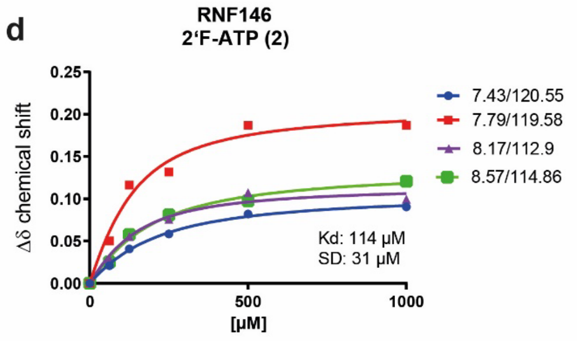

	7.43/120.55	7.79/119.58	8.17/112.9	8.57/114.86
Best-fit values				
Ptot	= 100	= 100	= 100	= 100
Kd	152	79.9	86.8	136
Bmax	0.107	0.209	0.116	0.136

e

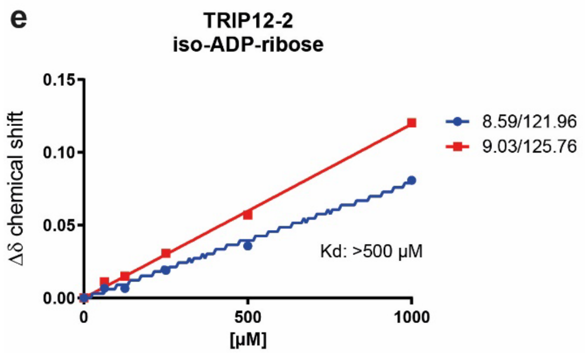

	8.59/121.96	9.03/125.76
Best-fit values		
Ptot	= 70.00	= 70.00
Kd	5638465533	~ 892518795
Bmax	445657	~ 106514

f

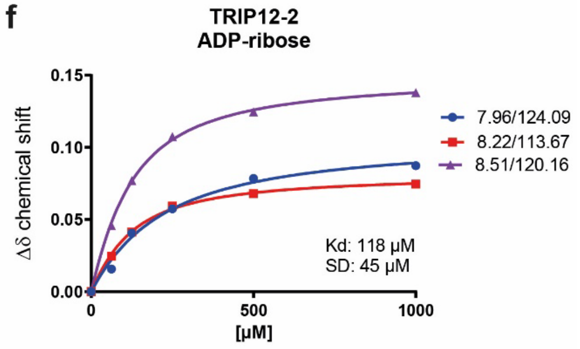

	7.96/124.09	8.22/113.67	8.51/120.16
Best-fit values			
Ptot	= 70.00	= 70.00	= 70.00
Kd	182.2	86.36	86.58
Bmax	0.1065	0.08187	0.1506

Figure S5: 15N-HSQC-NMR titration curves of RNF146 and TRIP12-2. (a-f) For the titration curve the ligand concentrations are plotted on the x-axis and the Δδ chemical shifts on the y-axis. The maximal plotted concentrations are up to compound saturation. The Δδ chemical shifts of one to four different peaks are followed of a single titration experiment. The position of the individual peak on the ^{1}H- and ^{15}N-scale of a ^{1}H-^{15}N-HSQC spectrum is labeled in ppms in the legend. Below the titration curve is a table with the position of the peaks in the column headers and the protein concentration in the experiment (Ptot), the dissociation constant (K_d) in µM and the maximum specific binding (Bmax) of each of the analyzed peaks in the rows. The titration curve of **(a)** ADPr, **(b)** ATP **(c)** 2F-ATP **(1)**, **(d)** 2'F-ATP **(2)** to RNF146 and (**e**) *iso*-ADPr and **(f)** ADPr to RNF146 is plotted. NMR K_d values originate from distinct samples (n=1) measured for each concentration, mean K_ds obtained from curves of selected cross peaks ± standard deviations.

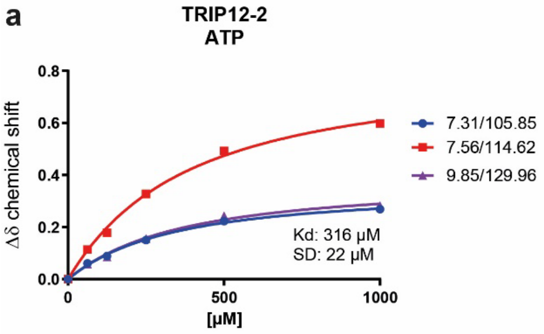

	7.31/105.85	7.56/114.62	9.85/129.96
Best-fit values			
Ptot	= 70.00	= 70.00	= 70.00
Kd	288.9	342.5	317.4
Bmax	0.3540	0.8275	0.3866

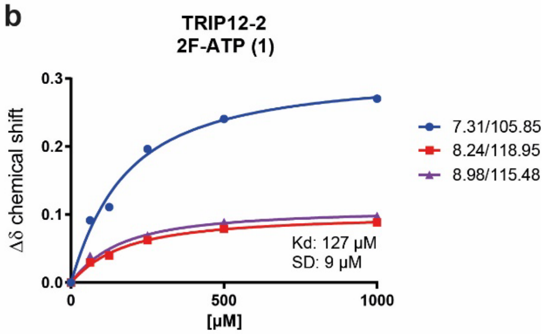

	7.31/105.85	8.24/118.95	8.98/115.48
Best-fit values			
Ptot	= 70.00	= 70.00	= 70.00
Kd	135.9	129.0	115.1
Bmax	0.3119	0.1009	0.1099

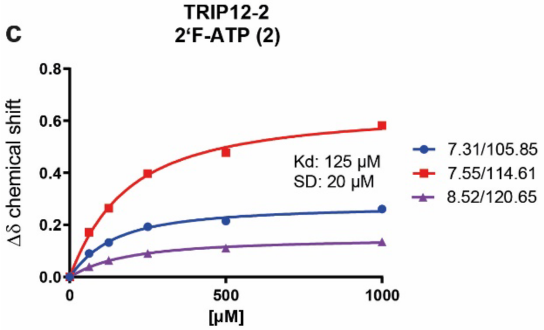

	7.31/105.85	7.55/114.61	8.52/120.65
Best-fit values			
Ptot	= 70.0	= 70.0	= 70.0
Kd	95.9	139	139
Bmax	0.278	0.654	0.151

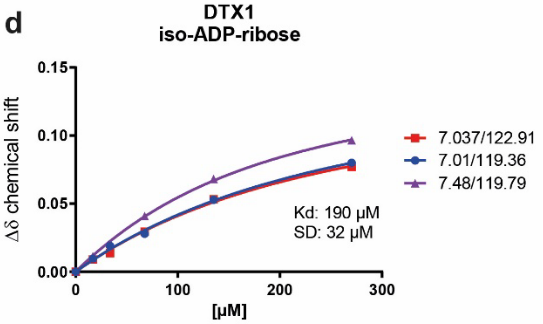

	7.01/119.36	7.037/122.91	7.48/119.79
Best-fit values			
Ptot	= 70.00	= 70.00	= 70.00
Kd	220.1	205.5	145.2
Bmax	0.1550	0.1461	0.1588

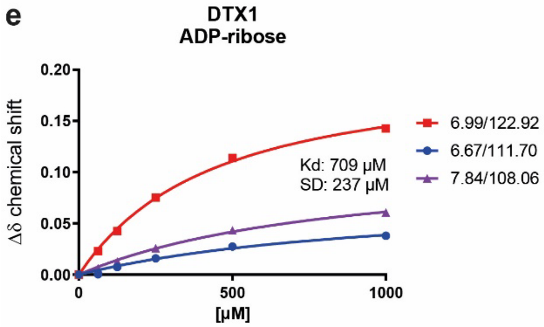

	6.67/111.70	6.99/122.92	7.84/108.06
Best-fit values			
Ptot	= 70.0	= 70.0	= 70.0
Kd	947	386	793
Bmax	0.0767	0.203	0.112

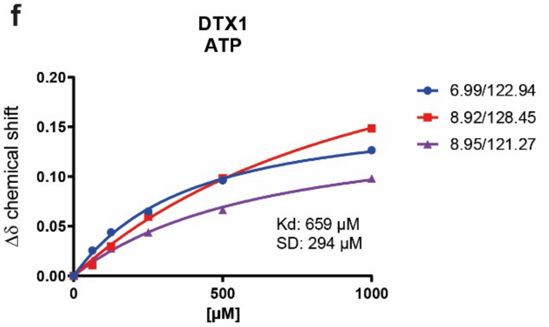

	6.99/122.94	8.92/128.45	8.95/121.27
Best-fit values			
Ptot	= 70.0	= 70.0	= 70.0
Kd	337	1048	592
Bmax	0.170	0.311	0.157

Figure S6: ^{15}N-HSQC-NMR titration curves of TRIP12-2 and DTX1. (a-b) For the titration curve the ligand concentrations are plotted on the x-axis and the Δδ chemical shifts on the y-axis. The maximal plotted concentrations are up to compound saturation. The Δδ chemical shifts of one to four different peaks are followed of a single titration experiment. The position of the individual peak on the ^{1}H- and ^{15}N-scale of a ^{1}H-^{15}N-HSQC spectrum is labeled in ppms in the legend. Below the titration curve is a table with the position of the peaks in the column headers and the protein concentration in the experiment (Ptot), the dissociation constant (K_d) in µM and the maximum specific binding (Bmax) of each of the analyzed peaks in the rows. The titration curve of **(a)** ATP, **(b)** 2F-ATP **(1)**, **(c)** 2'F-ATP **(2)** to TRIP12-2 and **(d)** *iso*-ADPr, **(e)** ADPr and **(f)** ATP to DTX1 is plotted. NMR K_d values originate from distinct samples (n=1) measured for each concentration, mean K_ds obtained from curves of selected cross peaks ± standard deviations

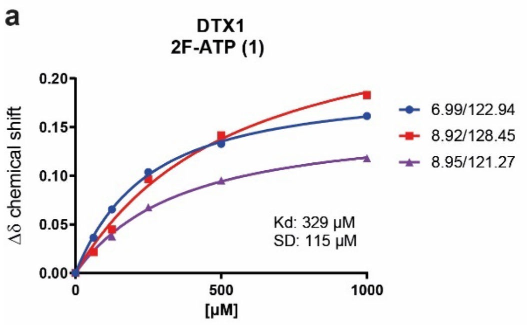

	6.99/122.94	8.92/128.45	8.95/121.27
Best-fit values			
Ptot	= 70.00	= 70.00	= 70.00
Kd	199.6	479.8	307.8
Bmax	0.1948	0.2793	0.1573

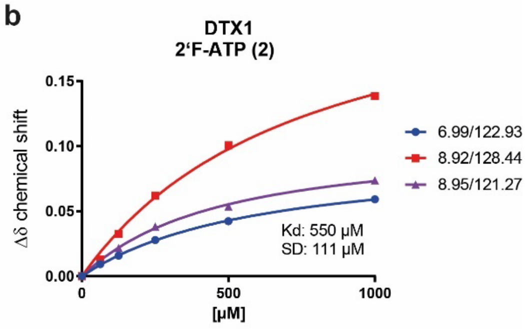

	6.99/122.93	8.92/128.44	8.95/121.27
Best-fit values			
Ptot	= 70.00	= 70.00	= 70.00
Kd	539.2	690.6	419.5
Bmax	0.09227	0.2408	0.1053

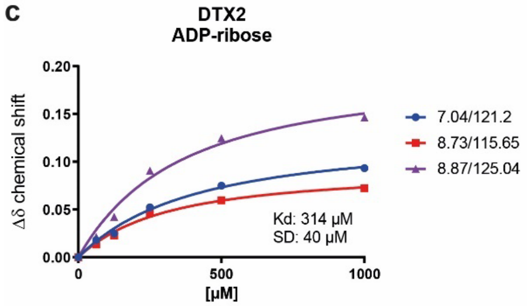

	7.04/121.2	8.73/115.65	8.87/125.04
Best-fit values			
Ptot	= 70.00	= 70.00	= 70.00
Kd	361.9	264.9	314.5
Bmax	0.1306	0.09369	0.2005

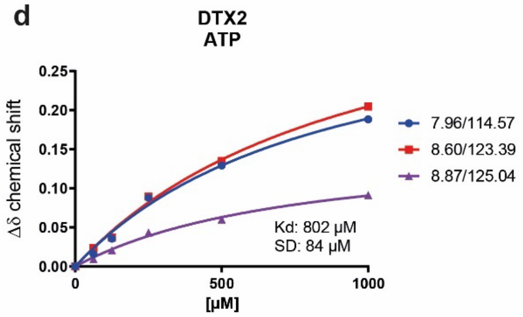

	7.96/114.57	8.60/123.39	8.87/125.04
Best-fit values			
Ptot	= 70.00	= 70.00	= 70.00
Kd	790.2	909.5	705.2
Bmax	0.3447	0.3977	0.1573

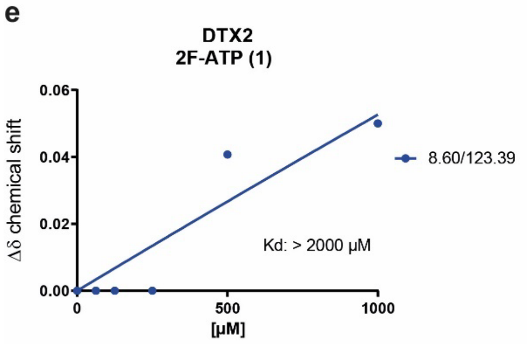

	8.60/123.39
Best-fit values	
Ptot	= 70.00
Kd	~ 35374
Bmax	~ 1.917

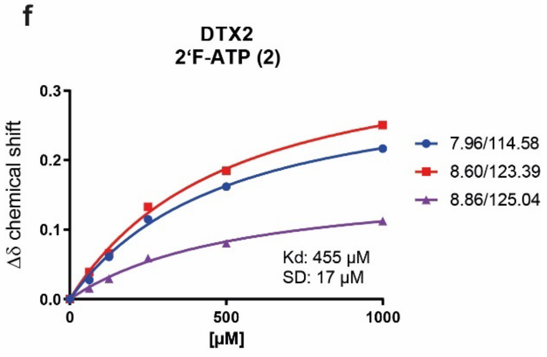

	7.96/114.58	8.60/123.39	8.86/125.04
Best-fit values			
Ptot	= 70.00	= 70.00	= 70.00
Kd	442.0	444.3	479.7
Bmax	0.3181	0.3672	0.1682

Figure S7: ^{15}N-HSQC-NMR titration curves of DTX1 and DTX2. (a-f) For the titration curve the ligand concentrations are plotted on the x-axis and the Δδ chemical shifts on the y-axis. The maximal plotted concentrations are up to compound saturation. The Δδ chemical shifts of one to four different peaks are followed of a single titration experiment. The position of the individual peak on the ^{1}H- and ^{15}N-scale of a ^{1}H-^{15}N-HSQC spectrum is labeled in ppms in the legend. Below the titration curve is a table with the position of the peaks in the column headers and the protein concentration in the experiment (Ptot), the dissociation constant (K_d) in μM and the maximum specific binding (Bmax) of each of the analyzed peaks in the rows. The titration curve of **(a)** 2F-ATP **(1)** and **(b)** 2'F-ATP **(2)** to DTX1 and **(c)** ADPr, **(d)** ATP, **(e)** 2F-ATP **(1)** and **(f)** 2'F-ADP **(2)** to DTX2 is plotted. NMR K_d values originate from distinct samples (n=1) measured for each concentration, mean K^ds obtained from curves of selected cross peaks ± standard deviations.

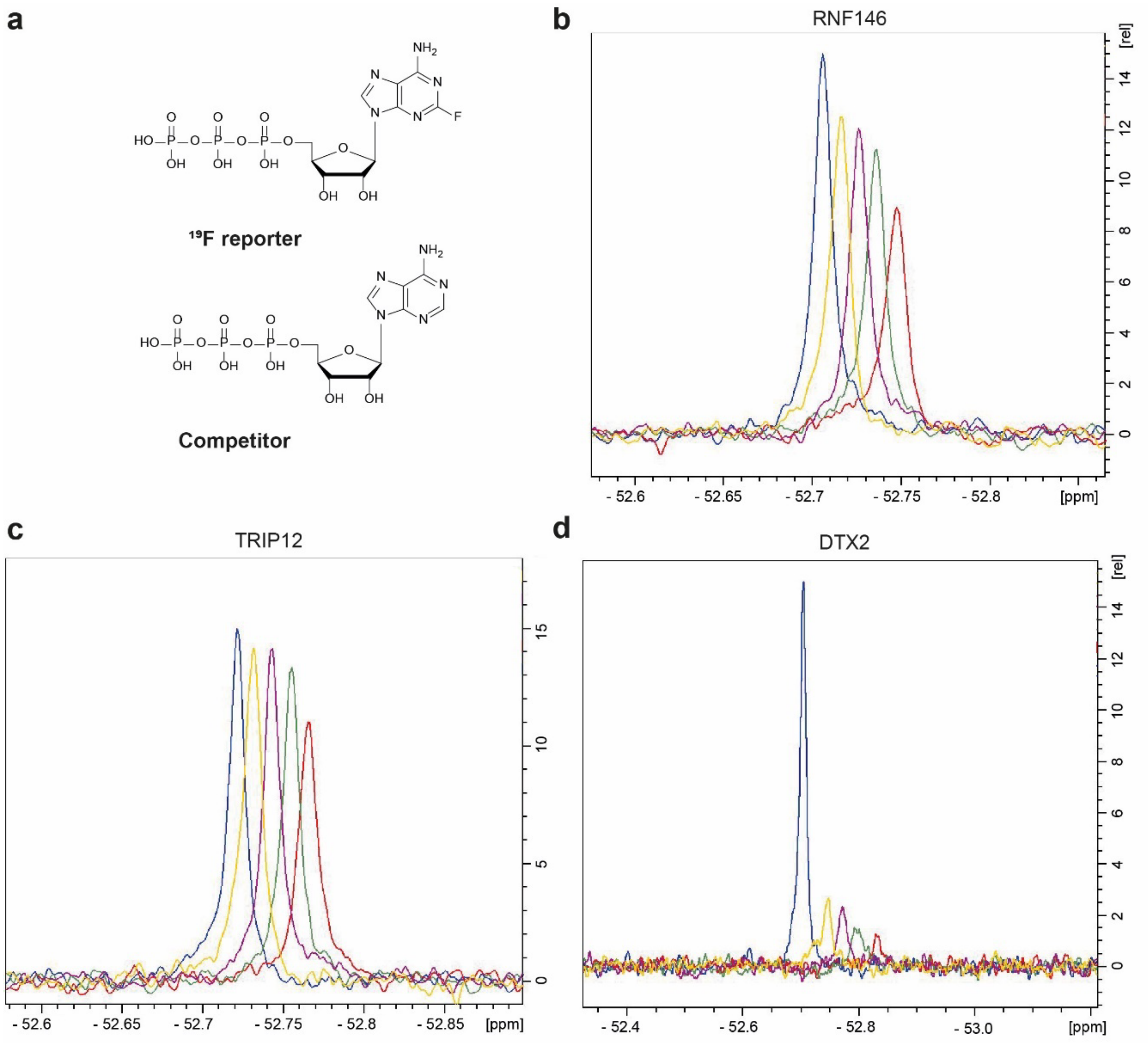

Figure S8: ^{19}F-NMR displacement assay with RNF146, TRIP12-2, and DTX2. (a) Chemical structure of ^{19}F reporter 2F-ATP **(1)** and competitor (ATP). **(b-d)** ^{19}F-Displacement assay with **(b)** RNF146, **(c)** TRIP12-2, and **(d)** DTX2. In red lines, the ^{19}F-NMR of 2F-ATP **(1)** reporter in the presence of protein is shown. In green, brown, and yellow, the titration of ATP at 250 mM, 500 mM, and 1000 mM concentration, respectively, to the protein in presence of 2F-ATP **(1)** is plotted. In blue lines, the ^{19}F-NMR of the reporter 2F-ATP **(1)** reporter without protein is shown.

Table S2: RMSD values for pairwise alignment of the reported structure of RNF146 WWE with *iso*-ADPr (PDB ID: 3V3L) against the nine structures reported in this study.

	**RNF146:*iso*-ADPr (3V3L)**
**HUWE1-ADPr** (chain D) (8RD7)	0.98 Å
**HUWE1-2'F-ATP (2)** (chain C) (8R7O)	1.07 Å
**TRIP12-ATP** (8TRE)	1.34 Å
**TRIP12-ADP** (9BKR)	1.41 Å
**DTX1-WWE1-ADP** (chain A) (8R6A)	1.27 Å
**DTX1-WWE2-ADP** (8R6B)	1.83 Å
**DTX1-ATP** (chain A) (8R5N)	1.29 Å
**HUWE1-Compound (3)** (chain C) (8RD0)	1.11 Å
**HUWE1-Compound (4)** (chain A) (8RD1)	1.15 Å

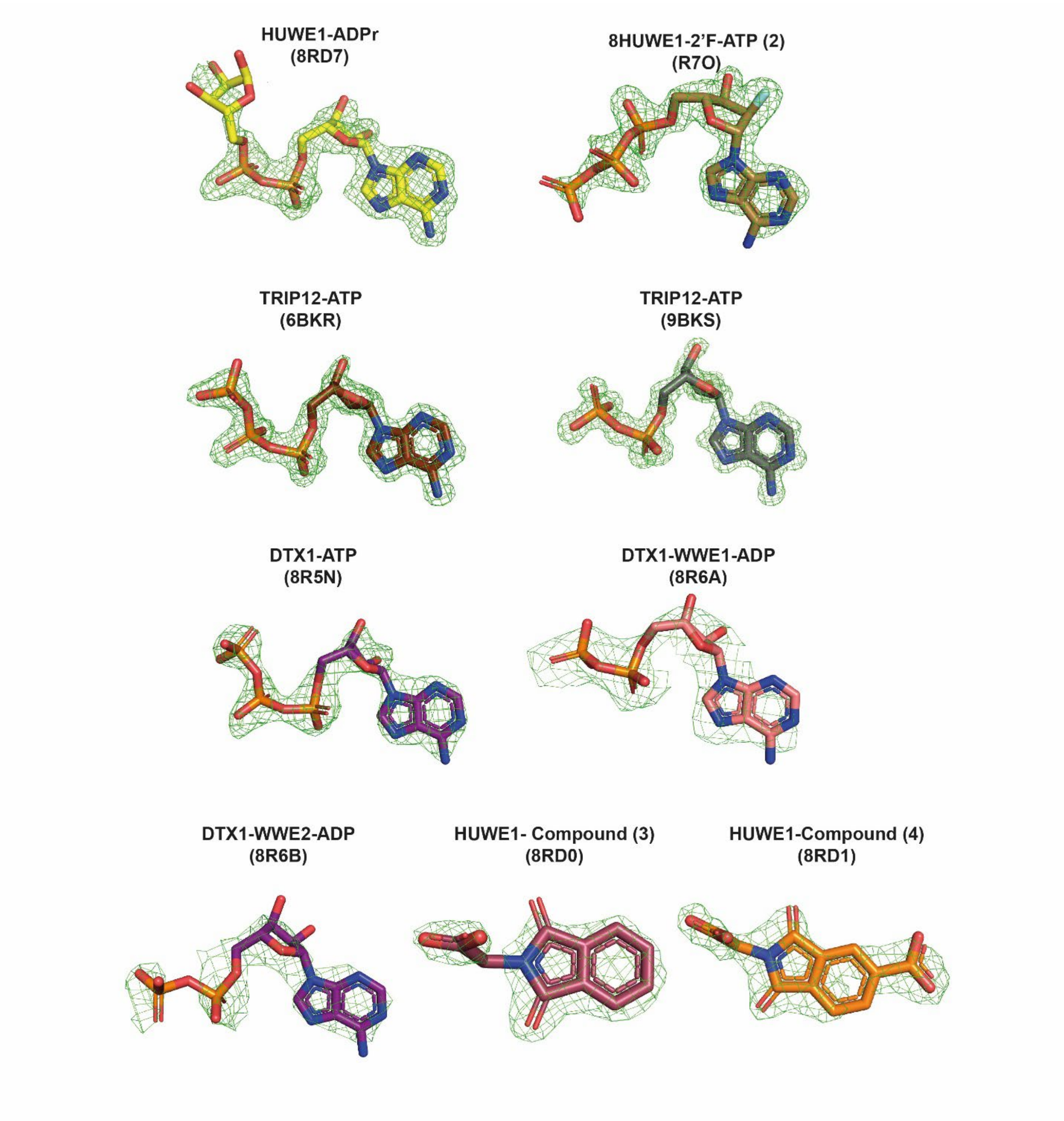

Figure S9: $F_o - F_c$ difference density (green mesh) calculated when the ligands of interest are omitted (contoured at 3σ except for DTX1-WWE1/2-ADP structures which are shown at a contour level of 2σ due to lower resolution of the respective structures).

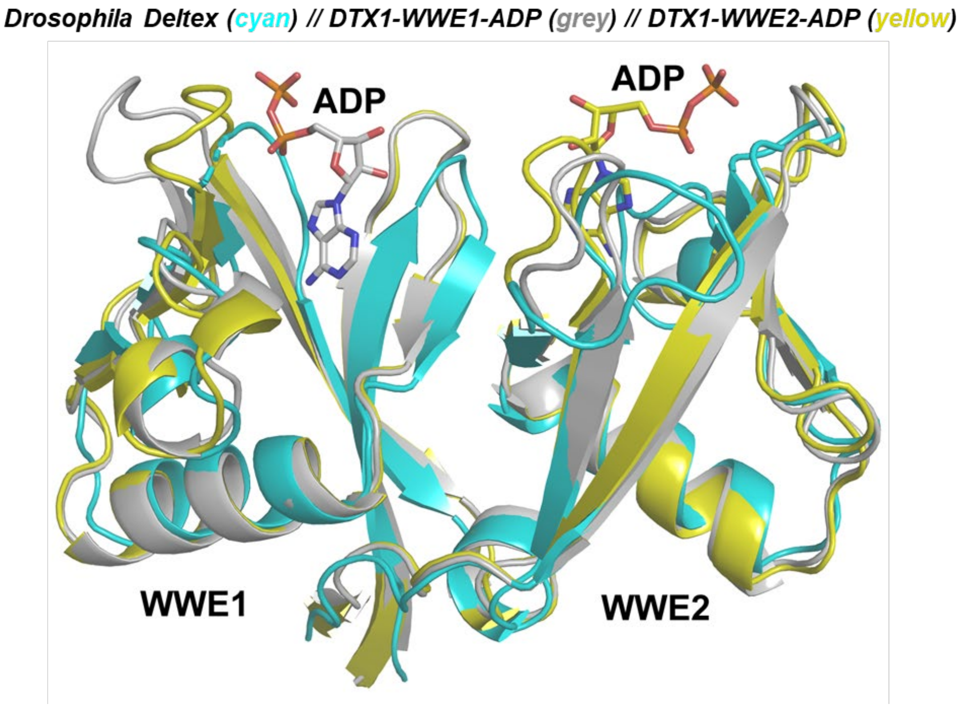

Figure S10: The fold conservation in the Deltex tandem WWE domains. A superposition of the reported *Drosophila* Deltex structure (2A90; cyan), the DTX1-WWE1-ADP structure (grey) and the DTX1-WWE2-ADP (yellow) showing fold conservation of both the WWE domains as well as the linking region. The ADP molecules (labeled) are rendered as sticks.

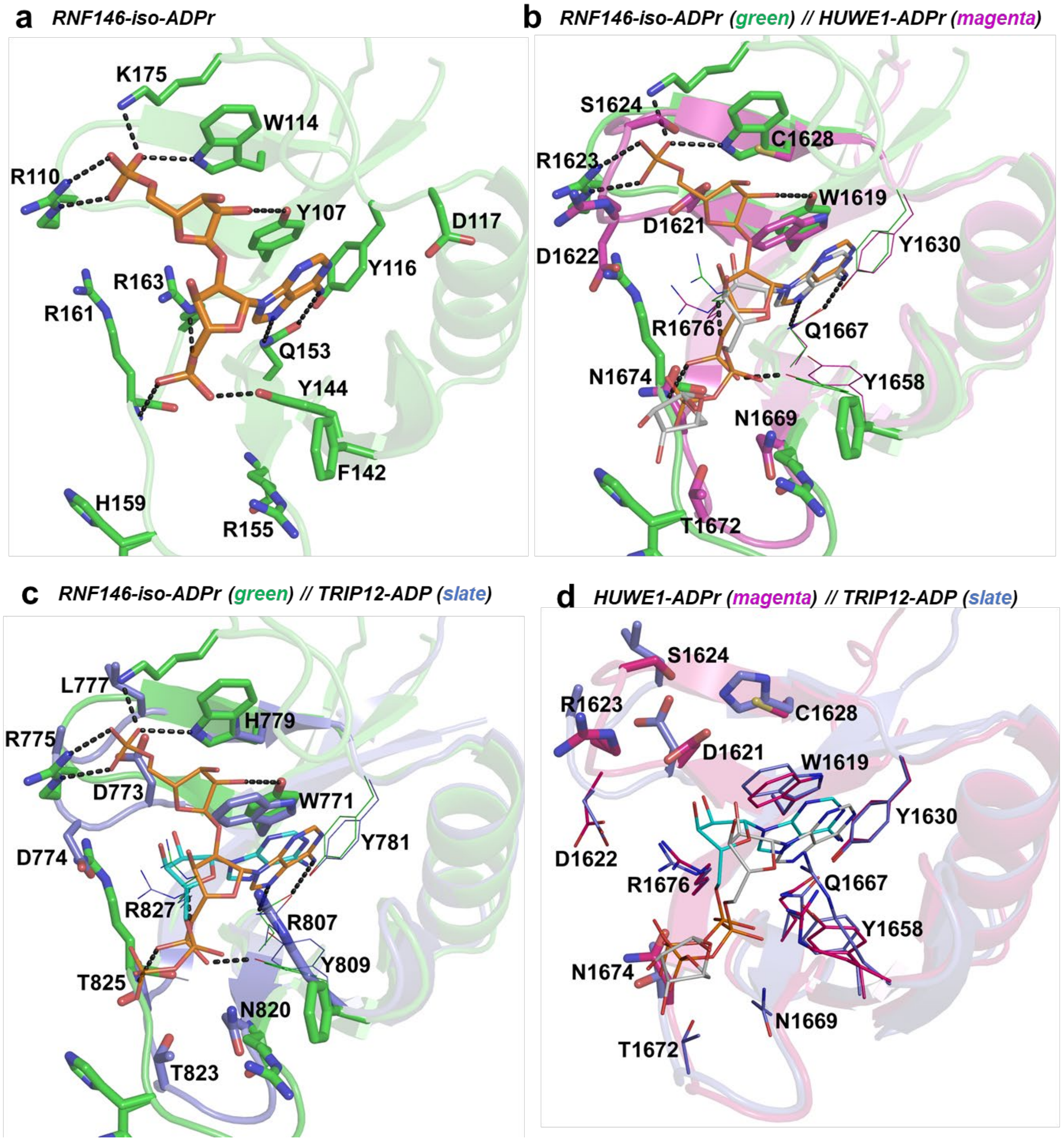

Figure S11: Comparisons of the natural WWE ligands binding sites. (a) The binding site of the *iso*-ADPr bound RNF146 WWE domain (PDB: 3V3L). *Iso*-ADPr is shown as orange sticks and potential hydrogen bonds are shown as black dashes. **(b)** Superposition of the HUWE1-ADPr WWE domain (magenta) with *iso*-ADPr bound RNF146 WWE domain (green; PDB: 3V3L), showing the differences in the binding mode of the two ligands as well as the residues that interact with each ligand to confer specificity. ADPr and *iso*-ADPr are depicted as grey and orange thin sticks, respectively. The conserved residues in the binding site are shown as lines while the non-conserved ones are shown as sticks. The binding site residues in the HUWE1-ADPr structure are labeled and possible interactions involving RNF146 and *iso*-ADPr are shown as black dashes. **(c)** Superposition of the TRIP12-ADP WWE domain (slate blue) with the RNF146-*iso*-ADPr WWE domain (green). ADP and *iso*-ADPr are rendered as cyan and orange thin sticks respectively. Conserved residues in the vicinity of the ligands are shown as lines while non-conserved ligand-interacting residues are shown as sticks. The binding site residues in the TRIP12-ADP structure are labeled and the possible interactions of RNF146 with *iso*-ADPr are shown as black dashes. **(d)** A superposition of ADPr-bound HUWE1 WWE domain (magenta) and ADP-bound TRIP12 WWE domain (slate blue). ADPr is shown as grey lines, ADP as cyan lines, the conserved ligand-interacting residues as lines and the non-conserved residues as sticks.

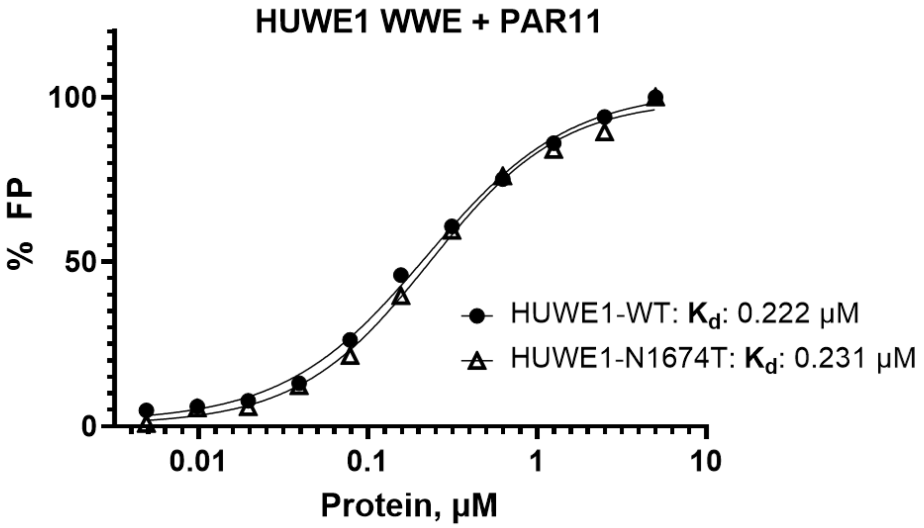

Figure S12: FP-based PAR11-mer direct_binding to the HUWE1 wildtype (HUWE1-WT) and HUWE1 N1674T mutant (HUWE1-N1674T) WWE domains. The fluorescence polarization percentage (% FP) of the reference is plotted as a function of HUWE1 protein concentration in μM using the logarithmic scale. The experiments were conducted in distinct triplicates (n=3) in 1 experimental repeat. The plotted values represent the averages from the 3 replicates.

WWE1 in DTX1-WWE1-ADP (grey) // WWE2 in DTX1-WWE2-ADP (yellow)

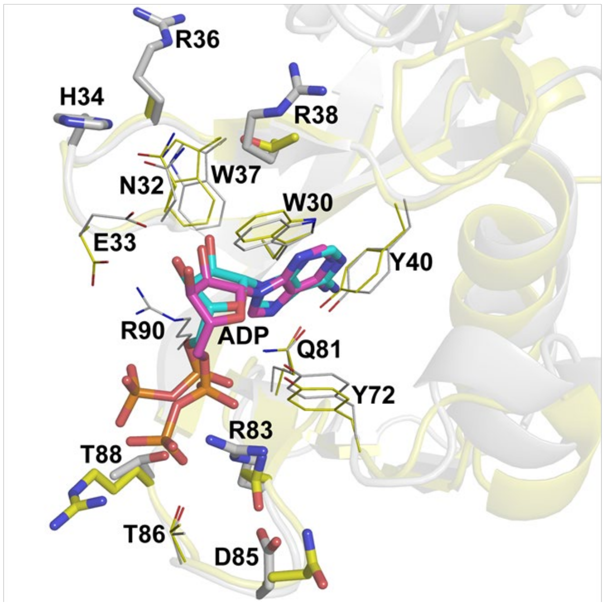

Figure S13: Comparison of the ADP-bound DTX1 WWE domains. A superposition of ADP bound DTX1 WWE1 (DTX1-WWE1-ADP, grey) and WWE2 (DTX1-WWE2-ADP, yellow) domains, highlighting amino acid differences in the binding sites between the two domains and the binding mode of the bound ADP molecules. ADP in the WWE1 domain is shown as cyan lines while the one in the WWE2 domain is shown as magenta lines. The conserved residues between the two binding sites are rendered as lines, while the non-conserved ones are depicted as sticks.

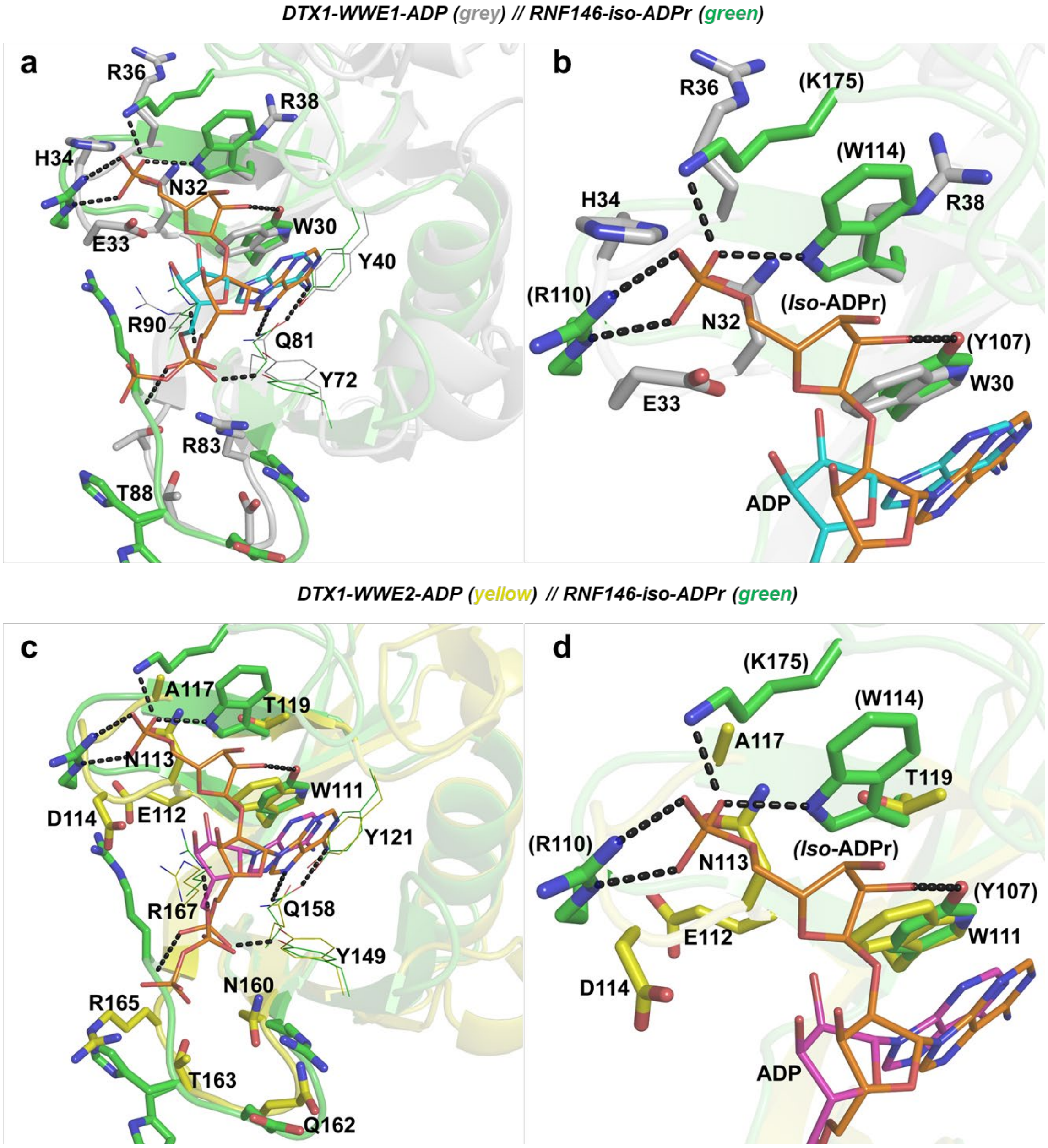

Figure S14: Comparisons of the ligand binding sites of RNF146-*iso*-ADPr and ADP-bound DTX1 WWE domains. **(a)** A superposition of the *iso*-ADPr bound RNF146 WWE domain (green; PDB: 3V3L) and the ADP bound DTX1 WWE1 domain (grey; DTX1-WWE1-ADP). *Iso-ADPr* is depicted as thin orange sticks while ADP is shown as thin cyan sticks. The conserved residues in the binding site are shown as lines while the non-conserved ones are shown as sticks. Residues of the DTX1-WWE1-ADP WWE domain are labeled and possible interactions involving RNF146 and *iso*-ADPr are shown as black dashes. **(b)** A superposition of the same structures in panel (a) showing the differences in the binding site of the distal ribose and phosphate groups of *iso*-ADPr. The binding site residues of both structures are labeled (RNF146-*iso*-ADPr numbers are in brackets) and possible interactions of *iso*-ADPr with RNF146 are shown as black dashes. **(c)** A superposition of the ADP bound WWE2 domain of DTX1-WWE2-ADP (yellow) and the iso-ADPr bound RNF146 WWE domain structure (green). The conserved residues in the binding site are shown as lines while the non-conserved ones are shown as sticks. Residues of the DTX1-WWE2-ADP WWE domain are labeled and possible interactions involving RNF146 and *iso*-ADPr are shown as black dashes. **(d)** A zoomed-in image of the the binding site of the distal ribose and phosphate groups of *iso*-ADPr in the superimposed structures in panel (c). The binding site residues of both structures are labeled (RNF146-*iso*-ADPr numbers are in brackets) and possible interactions of *iso*-ADPr with RNF146 are shown as black dashes.

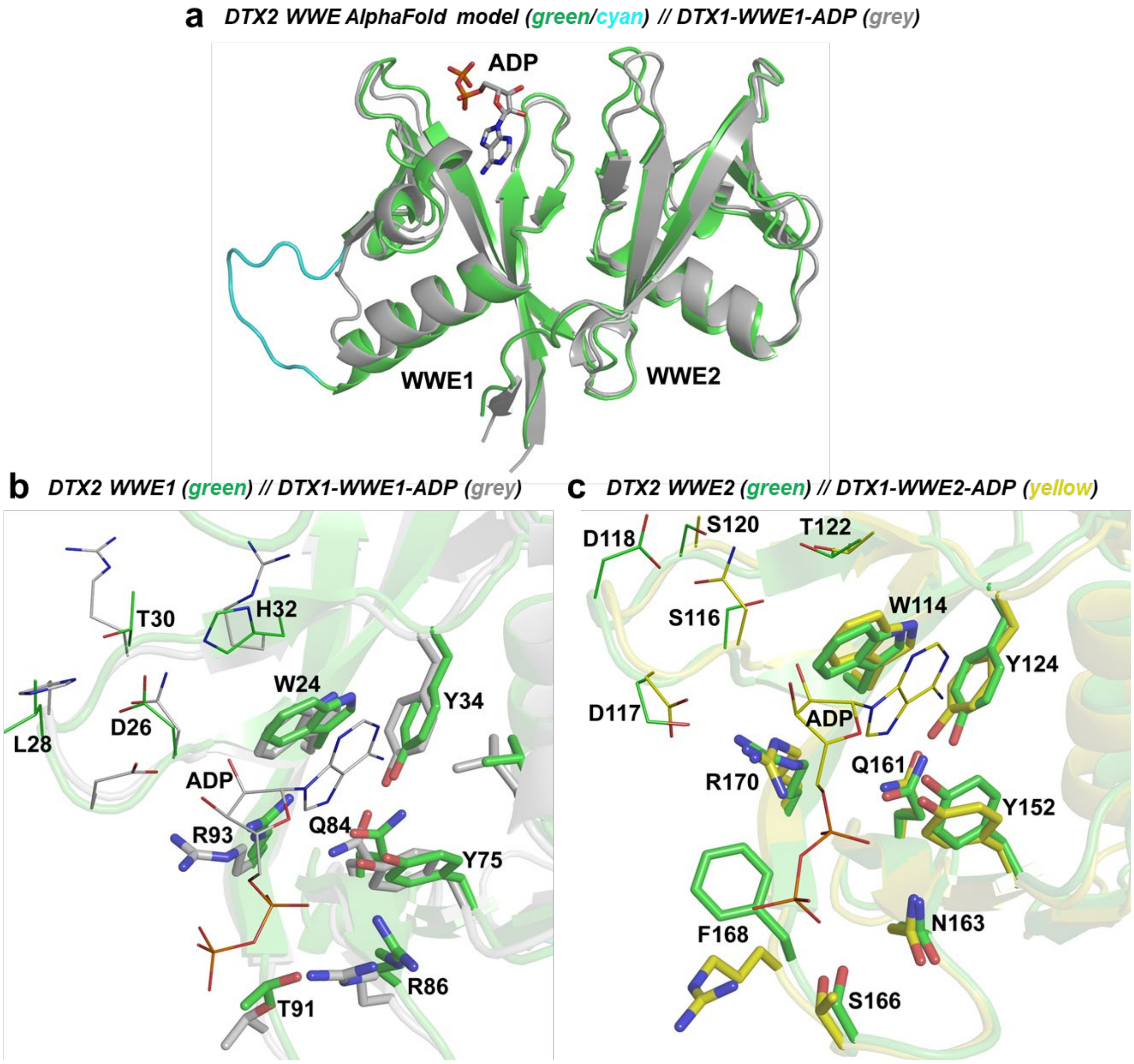

Figure S15: The AlphaFold predicted structure of the human DTX2 tandem WWE domains. (a) The predicted fold of the tandem DTX2 WWE domains (green) compared to the DTX1-WWE1-ADP complex structure with the ADP molecule (grey sticks) in the WWE1 domain (grey cartoons). **(b)** The binding site of the predicted DTX2 WWE1 structure (green) compared to that of the WWE1 domain (grey) in complex with ADP (grey lines) in the DTX1-WWE1-ADP structure. All the conserved residues in the DTX1 WWE1 binding site (sticks) are similarly positioned in the DTX2 WWE1 domain (also in sticks). Non-conserved residues are shown as lines and DTX2 WWE1 residues are labeled. **(c)** The binding site of the predicted DTX2 WWE2 domain (green) compared to that of the WWE2 domain (yellow) in complex with ADP (yellow sticks) in the DTX1-WWE2-ADP structure. The DTX2 WWE2 domain residues are labeled, conserved residues are shown as sticks and non-conserved residues are depicted as lines.

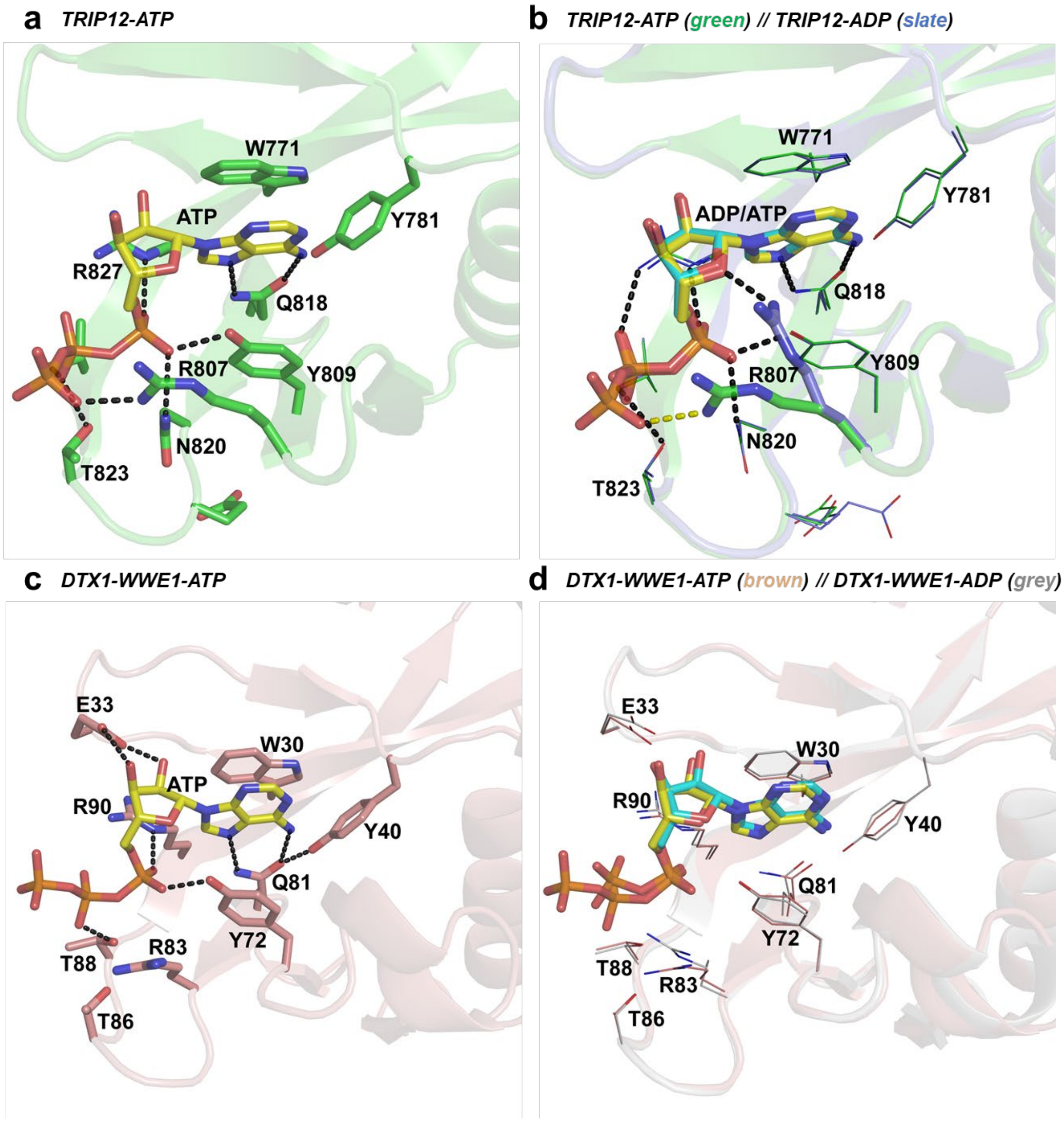

Figure S16: The binding of ATP to TRIP12 and DTX1 WWE domains. (a) The binding site of the TRIP12-ATP structure showing the binding mode and interactions of ATP (yellow sticks) with the protein. **(b)** A superposition of the TRIP12-ATP (green) with the TRIP12-ADP (slate blue) structures, showing the conservation of sidechain positions and orientation in the binding sites, except for Arg807 sidechain, which moves to interact with the terminal phosphate group of ATP. ADP in the TRIP12-ADP structure is depicted as cyan sticks, while ATP in the TRIP12-ATP structure is shown as yellow sticks. Potential hydrogen bonds are shown as dashes. **(c)** The binding site of the DTX1-WWE1-ATP structure showing the binding mode and interactions of ATP (yellow sticks) with the protein. **(d)** A superposition of the ATP bound DTX1 WWE1 (brown) and the ADP-bound DTX1 WWE1 domain (grey), showing the conservation of the sidechain positions despite the slight differences in the conformation of the ligands. ATP in the DTX1-WWE1-ATP structure is shown as yellow sticks while ADP in the DTX1-WWE1-ADP structure is depicted as cyan sticks. Potential hydrogen bonds are shown as dashes.

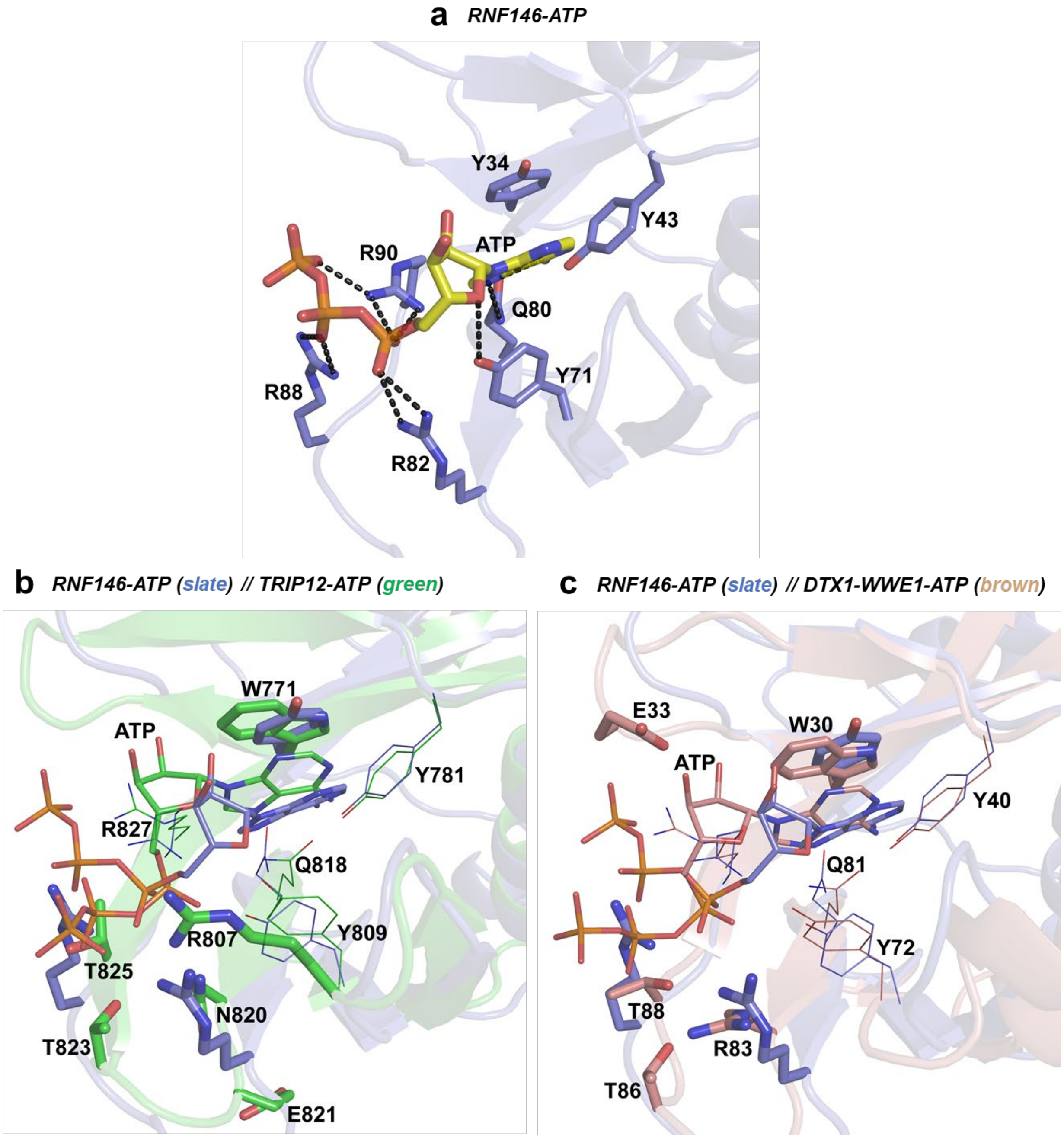

Figure S17: Comparisons of ATP binding to RNF146, TRIP12 and DTX1 WWE domains. (a) The binding site of the mouse RNF146-ATP NMR structure (PDB: 2RSF; ensemble 1) showing the binding mode and interactions of ATP (yellow sticks) with the protein. Potential hydrogen bonds are shown as black dashes. **(b)** A superposition of the RNF146-ATP (ensemble 1, slate) and the TRIP12-ATP (green) structures, highlighting the similarities and differences in the ATP binding mode. The ATP molecules are depicted as thin sticks in the same color as the respective proteins. Conserved residues are shown as lines, while the non-conserved ones are rendered as thick sticks. **(c)** A superposition of the ATP bound mouse RNF146 WWE domain (slate; ensemble 1) and the ATP bound DTX1 WWE1 domain (brown) structures. Conserved residues are rendered as lines, while non-conserved residues are shown as thick sticks. The ATP molecules are depicted as thin sticks in the same color as the respective proteins.

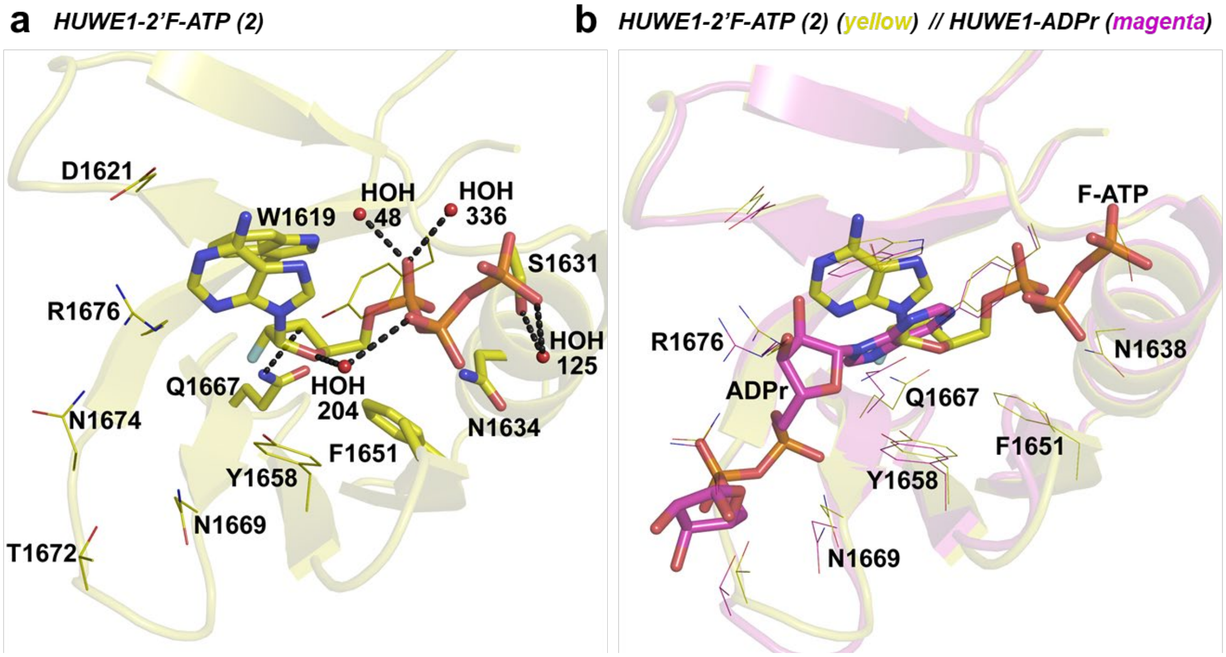

Figure S18. The binding of 2'F-ATP (2) to HUWE1 WWE domain. (a) The binding site of 2'F-ATP **(2)** bound HUWE1 WWE domain structure showing the binding mode and interactions of 2'F-ATP **(2)** (yellow sticks) with the protein. **(b)** A superposition of the HUWE1-ADPr (magenta) and the HUWE1-2'F-ATP **(2)** (yellow) structures, highlighting the large differences in the binding modes between ADPr (magenta sticks) and 2'F-ATP **(2)** (yellow sticks).
