# Supplementary material for: A ligand discovery toolbox for the WWE domain family of human E3 ligases
Source: Commun Biol. 2024 Jul 24;7:901. doi: 10.1038/s42003-024-06584-w (PMC11269756; doi:10.1038/s42003-024-06584-w)
Supplement: Supplementary file 4 — Reporting summary [file 42003_2024_6584_MOESM4_ESM.pdf]

nature portfolio

Corresponding author(s): Jark Böttcher

Last updated by author(s): 28.06.2024

Reporting Summary

Nature Portfolio wishes to improve the reproducibility of the work that we publish. This form provides structure for consistency and transparency in reporting. For further information on Nature Portfolio policies, see our Editorial Policies and the Editorial Policy Checklist.

Statistics

For all statistical analyses, confirm that the following items are present in the figure legend, table legend, main text, or Methods section.

n/a	Confirmed	
☐	☒	The exact sample size (n) for each experimental group/condition, given as a discrete number and unit of measurement
☐	☒	A statement on whether measurements were taken from distinct samples or whether the same sample was measured repeatedly
☒	☐	The statistical test(s) used AND whether they are one- or two-sided<br>*Only common tests should be described solely by name; describe more complex techniques in the Methods section.*
☒	☐	A description of all covariates tested
☒	☐	A description of any assumptions or corrections, such as tests of normality and adjustment for multiple comparisons
☒	☐	A full description of the statistical parameters including central tendency (e.g. means) or other basic estimates (e.g. regression coefficient) AND variation (e.g. standard deviation) or associated estimates of uncertainty (e.g. confidence intervals)
☒	☐	For null hypothesis testing, the test statistic (e.g. F, t, r) with confidence intervals, effect sizes, degrees of freedom and P value noted<br>*Give P values as exact values whenever suitable.*
☒	☐	For Bayesian analysis, information on the choice of priors and Markov chain Monte Carlo settings
☒	☐	For hierarchical and complex designs, identification of the appropriate level for tests and full reporting of outcomes
☒	☐	Estimates of effect sizes (e.g. Cohen's d, Pearson's r), indicating how they were calculated

Our web collection on statistics for biologists contains articles on many of the points above.

Software and code

Policy information about availability of computer code

Data collection	Diffraction data were collected on beamline 24-ID-C at the Advanced Photon Source in the Argonne National Laboratory and on beamline X10SA of the Swiss Light Source (Paul Scherrer Institute, Switzerland). NMR experiments were performed on a Bruker AVII 600 or 700 MHz spectrometer. FP was measured at room temperature using a BioTek Synergy 4 (BioTek, Winooski, VT)
Data analysis	15N-HSQC NMR spectra were recorded, and chemical shift perturbations were analyzed with Topspin 3.6 software from Bruker. Crystallographic data analysis was performed with autoPROC, XDS, CCP4, coot and autoBUSTER.The structures were validated using the Molprobity server68 and analyzed using UCSF Chimera69, and the molecular graphics images were rendered using PyMOL (The PyMOL Molecular Graphics System, Version 4.6 Schrödinger, LLC). FP data was analyzed with GraphPad Prism 9.5, GraphPad Software, Boston, Massachusetts USA.

For manuscripts utilizing custom algorithms or software that are central to the research but not yet described in published literature, software must be made available to editors and reviewers. We strongly encourage code deposition in a community repository (e.g. GitHub). See the Nature Portfolio guidelines for submitting code & software for further information.

Data

Policy information about availability of data

All manuscripts must include a data availability statement. This statement should provide the following information, where applicable:

- Accession codes, unique identifiers, or web links for publicly available datasets
- A description of any restrictions on data availability
- For clinical datasets or third party data, please ensure that the statement adheres to our policy

The model coordinates are deposited in the RCSB PDB under PDB IDs: 7UW7 (TRIP12-ADP), 8TRE (TRIP12-ATP), 8R7O (HUWE-2'F-ATP (2)), 8R6A (DTX1-ADP-WWE1), 8R6B (DTX1-ADP-WWE2), 8R5N (DTX1-ATP), 8RE1 (HUWE1-ADPr) 8RD0 (HUWE1-Compound (3)) and 8RD1 (HUWE1- Compound (4)). The source data behind the graphs in the paper can be found in the Supplementary Data files.

Research involving human participants, their data, or biological material

Policy information about studies with human participants or human data. See also policy information about sex, gender (identity/presentation), and sexual orientation and race, ethnicity and racism.

Reporting on sex and gender	NA
Reporting on race, ethnicity, or other socially relevant groupings	NA
Population characteristics	NA
Recruitment	NA
Ethics oversight	NA

Note that full information on the approval of the study protocol must also be provided in the manuscript.

Field-specific reporting

Please select the one below that is the best fit for your research. If you are not sure, read the appropriate sections before making your selection.

☒ Life sciences ☐ Behavioural & social sciences ☐ Ecological, evolutionary & environmental sciences

For a reference copy of the document with all sections, see nature.com/documents/nr-reporting-summary-flat.pdf

Life sciences study design

All studies must disclose on these points even when the disclosure is negative.

Sample size	No experiments were analyzed were individual samples were used for interference about larger populations. In case sample size was >1, this was used to acces reproducibility
Data exclusions	NA
Replication	All attemts of replication of data shown in this study were succesfull
Randomization	No experiments requiring randomisation were performed
Blinding	No experiments requiring blinding were performed. data generated in this study is not considered to be biased by subjects

Reporting for specific materials, systems and methods

We require information from authors about some types of materials, experimental systems and methods used in many studies. Here, indicate whether each material, system or method listed is relevant to your study. If you are not sure if a list item applies to your research, read the appropriate section before selecting a response.

Materials & experimental systems

n/a	Involved in the study
☒	☐ Antibodies
☒	☐ Eukaryotic cell lines
☒	☐ Palaeontology and archaeology
☒	☐ Animals and other organisms
☒	☐ Clinical data
☒	☐ Dual use research of concern
☒	☐ Plants

Methods

n/a	Involved in the study
☒	☐ ChIP-seq
☒	☐ Flow cytometry
☒	☐ MRI-based neuroimaging

Plants

Seed stocks	NA
Novel plant genotypes	NA
Authentication	NA
